# Current Insights and Advancements in Head and Neck Cancer: Emerging Biomarkers and Therapeutics with Cues from Single Cell and 3D Model Omics Profiling

**DOI:** 10.3389/fonc.2021.676948

**Published:** 2021-08-17

**Authors:** Yashika Jawa, Pooja Yadav, Shruti Gupta, Sivapar V. Mathan, Jyoti Pandey, Ajay K. Saxena, Suneel Kateriya, Ashu B. Tiku, Neelima Mondal, Jaydeep Bhattacharya, Shandar Ahmad, Rupesh Chaturvedi, Rakesh K. Tyagi, Vibha Tandon, Rana P. Singh

**Affiliations:** ^1^Special Center for Molecular Medicine, Jawaharlal Nehru University, New Delhi, India; ^2^School of Computational and Integrative Sciences, Jawaharlal Nehru University, New Delhi, India; ^3^School of Life Sciences, Jawaharlal Nehru University, New Delhi, India; ^4^School of Biotechnology, Jawaharlal Nehru University, New Delhi, India

**Keywords:** head and neck (H&N) cancer, single cell analysis (SCA), organoid technology, 3D culture, omics analyses, therapeutics of HNC

## Abstract

Head and neck cancer (HNC) is among the ten leading malignancies worldwide, with India solely contributing one-third of global oral cancer cases. The current focus of all cutting-edge strategies against this global malignancy are directed towards the heterogeneous tumor microenvironment that obstructs most treatment blueprints. Subsequent to the portrayal of established information, the review details the application of single cell technology, organoids and spheroid technology in relevance to head and neck cancer and the tumor microenvironment acknowledging the resistance pattern of the heterogeneous cell population in HNC. Bioinformatic tools are used for study of differentially expressed genes and further omics data analysis. However, these tools have several challenges and limitations when analyzing single-cell gene expression data that are discussed briefly. The review further examines the omics of HNC, through comprehensive analyses of genomics, transcriptomics, proteomics, metabolomics, and epigenomics profiles. Patterns of alterations vary between patients, thus heterogeneity and molecular alterations between patients have driven the clinical significance of molecular targeted therapies. The analyses of potential molecular targets in HNC are discussed with connotation to the alteration of key pathways in HNC followed by a comprehensive study of protein kinases as novel drug targets including its ATPase and additional binding pockets, non-catalytic domains and single residues. We herein review, the therapeutic agents targeting the potential biomarkers in light of new molecular targeted therapies. In the final analysis, this review suggests that the development of improved target-specific personalized therapies can combat HNC’s global plight.

## Introduction

The origin of cancer is traced to the characteristic unresponsive cellular behavior towards signals that regulate survival, proliferation, differentiation, and eventual evasion of death (1). The research into the biological mechanisms of cancer progression has advanced our knowledge of disease biology, and new developments in effective anti-tumor therapies have generated a stream of possibilities and strategies to tackle a wide range of cancer types. Despite these advances, head and neck cancer (HNC) remains among the ten most common malignancies worldwide with higher rankings in developing countries (2). The HNCs are categorized by origin in the head, neck, or the upper aero-digestive tract including oral cavity, para-nasal sinuses, pharynx, larynx, cervical esophagus, thyroid, associated lymph nodes, soft tissues, and bone (3). A broad spectrum of tumors arising from different head and neck tissues are designated as HNC, with >90% of malignancies being squamous cell carcinomas (SCC) and its variants. Histopathologically, other tumor of the head and neck region include adenocarcinomas, sarcomas, anaplastic carcinoma, plasmacytoma, lymphomas, and malignant melanoma.

Approximately 75% of the cases of HNC worldwide can be associated with its classical causative agents; heavy tobacco or alcohol consumption (3, 4). Human papillomaviruses (HPV) are also important causative agents for the development of oropharyngeal tumor of the tonsils or the tongue basal area (5). HPV positive tumors exhibit better prognosis and show little correlation with tobacco and alcohol exposure unlike HPV negative tumors (6). Likely, other unknown factors could also play essential roles in tumorigenesis, tumor progression, and metastasis of HNC, such as alteration in microbial diversity and function, genetic polymorphisms in enzymes involved in alcohol and tobacco metabolism (7, 8) or, genetic predisposition as is in Li Fraumeni’s syndrome, Fanconi’s anemia and ataxia telangiectasia (9).

Though several drugs are presently in clinical trials (10, 11), most treatment strategies are hamstrung by limited patient response and the complex tumor microenvironment. Therefore, in-depth studies to elucidate the mechanism of action of drugs, and the challenges that cripple their efficacy, are necessary before devising new molecules with increased efficacy. Till very recently, predicting clone genotypes from tumor bulk sequencing of multiple samples was cardinal to the delineation of tumor profiles. Since drug-resistant clones develop throughout the tumor growth process, their presence often precedes a drug treatment regimen strategy; enabling single tumor cells to evade drug treatment camouflaged by their divergent profiles. Single-cell analysis, spheroids, organoids technology are emerging as solutions that can be exploited for effective treatment strategies by mapping individual genetic profiles of heterogeneous tumor cells.

## Heterogeneity: A Challenge in the Treatment of Head and Neck Cancer and Road Towards Solutions

Head and neck cancers, notorious for their heterogeneity and relapsing nature require an improved understanding and characterization in order to counter recurrence, resistance and disparities in therapeutic responses. This heterogeneity and anatomical diversity makes the treatment protocol a virtual nightmare and also demands linking of phenotypic assay data with clinical outcomes in order to optimize the treatment and translate benefits to the patients (12). Though the Cancer Genome Atlas has increased perception of inter-tumoral heterogeneity across scores of patients, the knowledge of intra-tumoral heterogeneity stays very rudimentary.

The conventional diagnostic techniques analyze the tumor population as a whole and, as a result, derive an inference which averages the effects of all different types of cells in the population. Until recently, genotypes were predicted using tumor sequencing from multiple and bulk samples (13). However, the average targeting of cancer is grossly inadequate and strategies are required to characterize individual cancer cells and subsequently optimize treatment regimens. The development of models that consider as well as provide the interactions with ECM and cells of the microenvironment (like cancer-associated fibroblasts (CAFs), myeloid derived suppressor cells (MDSCs) and immune cells like Th1, Th2, Treg cells & cytotoxic T cells, M1 & M2 macrophages, N1 & N2 neutrophils, natural killer cells (NK cells), dendritic cells etc.) (14) becomes necessary. These models necessarily require to mimic other *in vivo* conditions as well, such as hypoxia which is said to be responsible for stemness (15) and radio-resistance (16), both prominently seen in HNCs.

**Figure 1:**
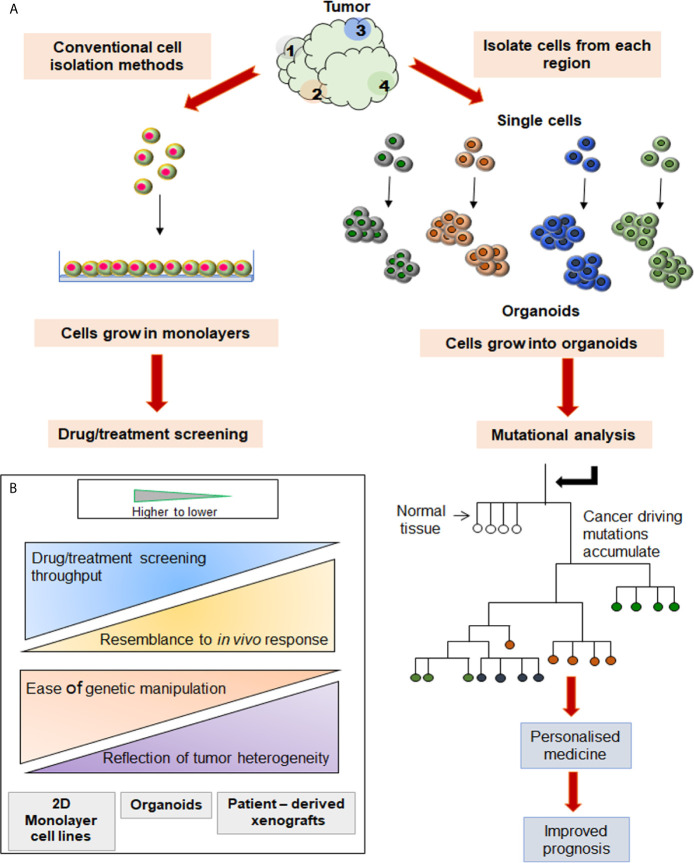
Comparison between two different cell culture systems initiated from the same source. **(A)** Cells isolated from various tumor sites are grown in 2D and 3D culture systems, and their trajectories are tabulated on the right and left side, respectively. Analysis of mutations in each organoid grown from a single cell may be used to construct the phylogenetic tree. **(B)** When compared to PDXs or slice cultures, the 3D cultures are amenable for easier manipulation, identification of heterogenous population, and high throughput screening (HTS).

The single-cell analytical methods and spheroids/organoid models are being found particularly useful in cancer biology and clinical oncology. Aiming to improve the understanding of two key areas, cancer research and, drug discovery, the latter provides suitable models to reproduce the tumor microenvironment while the former gives an accurate measure of cell properties and minimizes adulteration or approximation associated with bulk measurements. The conventional 2D cultures include growing transformed cells derived from tissues in monolayer cultures. Although characterized by easy maintenance and experimental modifications, the extended survival of cancer cell lines in these monolayer cultures allows for the development of undefined mutations and the consequent loss of parental cells’ genetic characteristics (17). Also, the cellular heterogeneity and tissue architecture found in tissues or tumor of their origin is lacking in 2D cultures. On the other hand, organoid and spheroid cultures can mimic or recapitulate the tumor microenvironment signaling by partially permitting vital cell-cell contacts, cell signaling, and cell-ECM interactions. Their higher physiological relevance, susceptibility to manipulation of niche components, signaling pathways and genome editing, makes them an important bridge between 2D culture and *in vivo* animal models (**Figure 1**).

**Figure 2:**
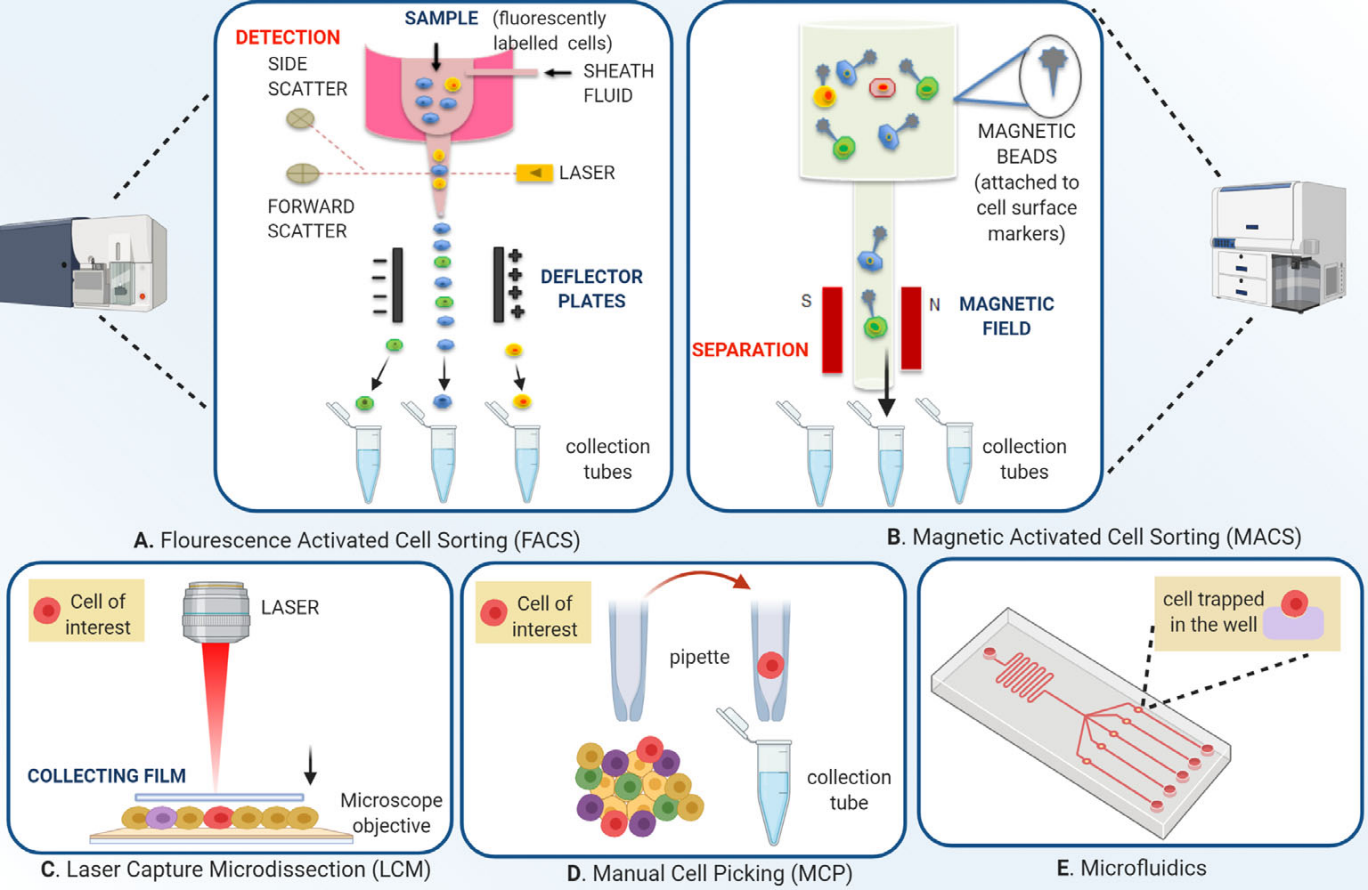
Overview of single-cell isolation technologies. **(A)** Schematic of fluorescence-activated cell sorting; FACS employs two separate techniques, streamlining the fluorescently labeled cells to pass through a micro-spectrophotometer one cell at a time, and a second to record the emission of the signal. The signals are based on cell dimensions, coarseness, and fluorescence. The technique allows both qualitative and quantitative analysis of a cell population. After the initial sample preparation, the cell suspension is passed in a monolayer in a manner that each cell is subjected to exposure by a laser which permits the fluorescent labels to be identified by the instrument. The instrument applies a charge depending on the nature of the cell, which deflects a droplet containing the cell of interest from the entire flow. This charged droplet is then collected by collection tubes. **(B)** Magnetic-activated cell sorting differs from FACS in the way that instead of fluorophore tagged labels, this technique uses magnetic bead conjugated with antibodies, streptavidin, lectins, or enzymes. The cells are channelized under an applied magnetic field that allows non-conjugated cells to pass freely. The magnetic bead conjugated cells are then eluted by turning the magnetic field off. Separation can be both positive and negative. Positive separation employs a technique where the cells of interest are conjugated with the magnetic beads. **(C)** Laser capture micro-dissection uses an inverted microscope, an infrared or ultraviolet laser, and an extraction system. After visual identification of the cell of interest, through a user-defined pattern, the laser cuts the cell from the population. Various extraction methods are used, one of them being the laser activating an adhesive on a thin film kept over the tissue, which in turn sticks to the cell of interest, and the cell can be removed by picking up the film. **(D)** Manual cell picking also employs an inverted microscope, but instead of lasers, automated micropipettes are used for the cell extraction. MCP’s main advantage over LCM is that live cell cultures can be isolated, in contrast to fixed cells in LCM and **(E)** A microfluidic device depends on the capture of single cells from the suspension so well diluted that the probability of one cell going into one well is maximum. The microwell technique can accommodate single-cell imaging along with analysis. Automated devices streamline the cells in a microflow and sort the cells according to specific properties like size, charge, or ligand affinity into different populations.

In view of the above, it is reasonable to hypothesize that the organoid and single cell technologies have applicative potential in HNC where identifying, understanding and, addressing the tumor heterogeneity is the primary concern. These technologies can be applied either independently or in combination to discover novel biomarkers and specific molecular targets. Subsequently, the information so retrieved can be supportive of streamlining the drug development procedure (**Figure 1**). The approaches used for single single-cell isolation vary from targeting either their physical or biological characteristics (**Figure 2**). The physical characteristics like electric charges, density, size, and flexibility, are exploited by the microchip-based capture platforms, membrane filtration, and density gradient centrifugation. On the contrary, the cells’ biological characteristics such as cell surface markers, size, granularity help in single-cell isolation *via* affinity chromatography, fluorescence-activated cell sorting (FACS), and magnetic–activated cell sorting (MACS) methods (18). To characterize the heterogeneity in tumor mass and microenvironment, single-cell separation and culturing techniques are significant. These methods not only utilize the physical properties of cells but have added advantage of being label-free techniques. Thus, single cell-sorting and omics analysis techniques have become the backbone of current investigations in the direction of personalized treatment in all forms of cancer including HNC. The single-cell technologies operate on the dual platforms of ‘single-cell separation’ and ‘single-cell analysis’. These technologies are certainly warranted for detection and analysis of intra-tumor heterogeneity (ITH) and decipher the mechanisms of tumor metastasis, investigate omics alterations, and discover precise treatment strategies (19).

**Figure 3:**
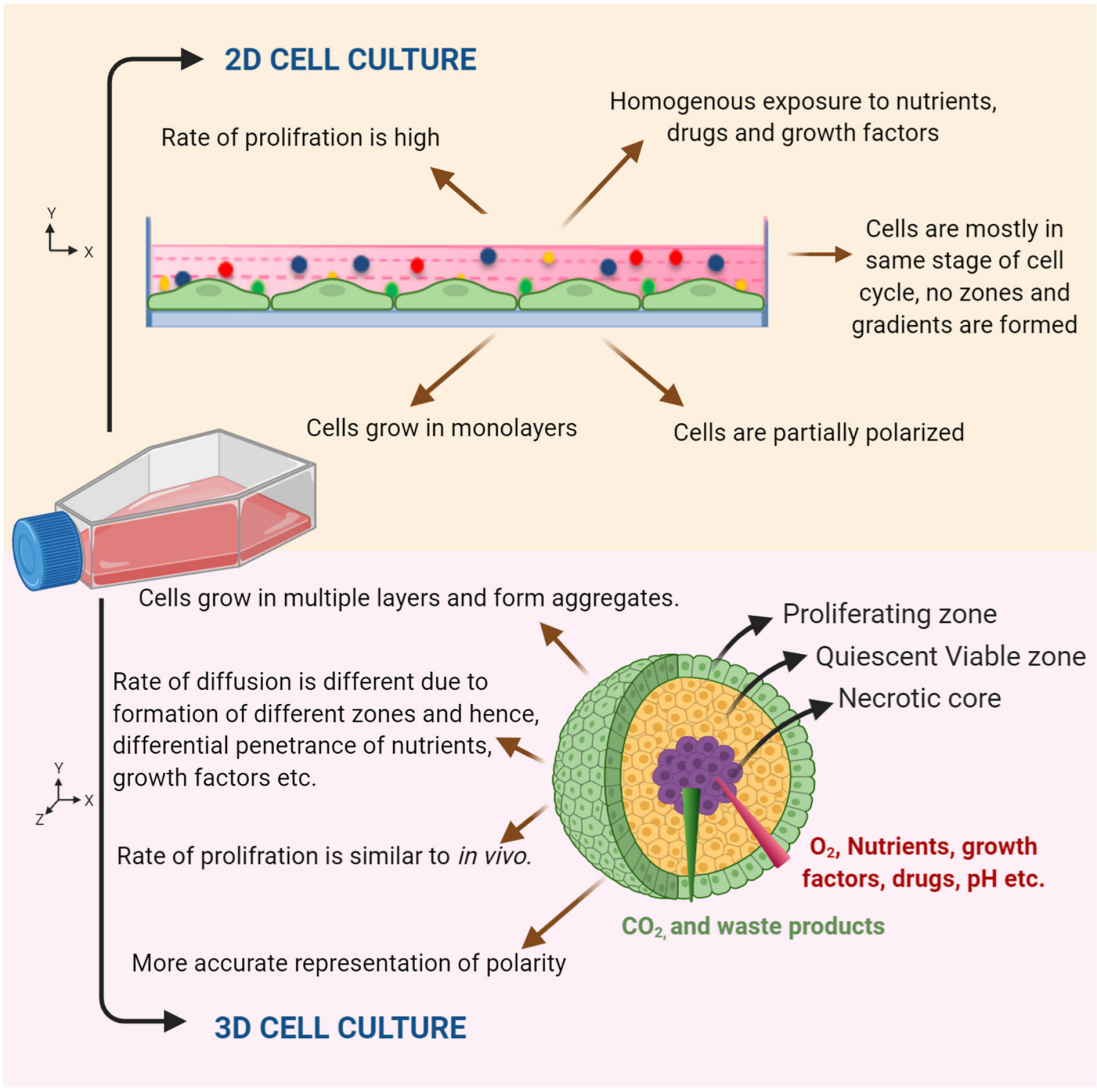
Characteristic features of 2D and organoid culture. Schematic representation showing cells grown in 2D monolayer culture and in 3D culture. An organoid mimics the tumor microenvironment by forming different zones (viz. proliferating zone, quiescent viable zone, and necrotic core) and gradients, which gives a realistic response compared to 2D monolayer cultures.

Several gene expression, metabolic and drug response based studies have reiterated the importance of 3D culture, as it mimicks in vivo cell environment in a better manner as compared to 2D culture (**Figure 3**). A study by Shah et al. characterized head and neck cancer organoids metabolically. The cell metabolism was analyzed by measuring the intrinsic fluorescence of NAD(P)H and FAD on a single cell level before and after treatment. The redox ratios of the organoids were measured in response to different drug treatments. Therefore, the study suggested the use of organoids as a complementary tool to perform rapid comparisons between treated and non-treated samples, to observe metabolic response to drugs and to characterize heterogeneity (20). Another study by Tanaka et al. used CTOS (Cancer tissue originated spheroids) method to establish HNC organoids. The study also characterized marker expression profile in spheroids in comparison to the original tumor cell, finally showing similar marker expression of cancer stem cell to *in vivo*. Exposure to drugs like cisplatin and docetaxel was able to accurately define drug sensitivity *in vivo* (21). The Driehuis et al. provided a standardized protocol for generation of HNC organoids using patient tumor samples and their subsequent use in drug screenings. This allowed comparison of differential drug responses in different patients. The study also floats the idea that organoids may potentially predict patient clinical responses (22). The same group in a previous study primarily focused on using 3D models for testing *in vitro* targeted PDT (photodynamic therapy). Since EGFR is primarily targeted in PDT, its expression levels were compared in organoids to that of cell lines used previously. The levels in organoids recapitulated both tumor and normal patient samples. In fact, organoids from tumor were found to be more sensitive to PDT than their corresponding normal/wild type tissues. This suggests that the therapy may prove more significant as it will leave surrounding normal epithelia of tumor unaffected. Therefore, also highlighting the use of EGFR as a major molecular target in HNC which is already suggested by multiple studies (23). Multiple studies have also reiterated that organoids are not relevant only because they grow in 3D spatial arrangement mimicking *in vivo* conditions but also because they capture distinct behaviors of respective tumor they arise from (24).

Co-culture systems in the form of 3D organoid models are gaining more attention recently and are being used for assessing the anticancer effects. Within the tumor microenvironment, the cell-cell interactions between Cancer associated fibroblast (CAFs) and cancer cells contribute to carcinogenesis, *via* tumor initiation, progression and metastasis (25). Similarly, paracrine signaling between stromal and cancer cells is known to mutually stimulate proliferation and induction of drug resistance. **Table 1** discusses some of the model systems used in HNC related studies. Different models allow for customizations relevant to parameters under investigation and provide an edge over conventional techniques. For instance, to elucidate monocyte action, they can be cultured along with HNC cells (26–29). Similarly, fibroblasts and PBMCs can also be co-cultured with HNC cells for EGFR based studies (30) and for testing antibodies (31), respectively. Other studies have shown the role of TAMs (Tumor associated macrophages) and HDFs (Human Dermal Fibroblasts) in cancer stemness and invasion respectively (32, 33). Differential drug response towards EGFR targeting drugs is studied using CAFs (34). Different organoid model systems of HNC were established to explore ERK1/2 and Nanog signaling (35), HSV1 and HPV16 (36), invasiveness in cancer (37), drug screening (38), and other characteristic hallmarks (39). Hydrodynamic shuttling chip (HSC) is a microfluidics platform through which single-cell squamous carcinoma cells are separated and co-cultured with lymphatic endothelial cells to observe the motility and cell-cell communications (40).

**Table 1:** Different 3D models of HNC.

S.No.	3D culture models	Application
1	Malignant/benign HNC + Mucosa monocytes	IL-6 secretion and prediction of prognosis
		Mechanisms for monocyte activation
		Tumor-associated macrophages are a source of monocyte chemotactic protein (MCP-1) in HNC tumors.
2	HNC cell line + fibroblasts	Anti-EGFR monoclonal antibody
3	HNC cell line +PBMC	Trifunctional bispecific antibody catumaxomab
4	HNC spheroids + tumor Associated macrophages (TAMs)	CD44 signaling and mechanistic link of TAMs in cancer stemness
5	HNC OECM-1 cell line + human dermal fibroblasts (HDFs)	Cancer cell invasion in collagen microenvironment
6	HNC spheroids + CAFs	Differential drug response to cetuximab and mTOR inhibitor
7	HNC spheroids	Role of ERK1/2-Nanog signaling in head and neck cancer stemness
8	Oral mucosal Organoids and HNC patient-derived tumoroids	To study oral mucosal pathology by infecting with HSV1 and HPV16 and HNC patient-derived tumoroids utilized for drug screening and personalized medicine.
9	HNC Multicellular tumor spheroid (MCTS)	Drug screening

State of the art methods for culturing 3D cells are classified on the basis of source materials used, 3D environment, kind of scaffold and the types of cultures generated. Various methods have evolved with date and used for 3D cell culturing are scaffold-dependent methods (41) *viz*. hydrogel method (42), agarose coating method (43); and scaffold-independent methods (44) like hanging drop method, rotary cell culture system, micropatterning, microfluidics (45), low-attachment plates method (46), magnetic-cell leviation (47). Organoids derived from single cells can generate enough biomass for investigating tumor heterogeneity at the single cell level (**Figure 1**). Patient-derived organoids (PDOs) are particularly useful as models for specific diseases or infections, which otherwise are difficult to generate or probe in animal models.

Despite many applications 3D cultures cannot mimic *in vivo* growth factor, biomechanical forces etc. Thus, organoids, in spite of their potential as near-physiological cell culture models, are difficult to culture with unknown or unfamiliar niche or growth factors, and necessitate high technical skill and elaborate experimental set up in most cases. In addition to these, the field of HNC still requires more comprehensive studies using organoid technology as the literature available is less compared to other cancers like breast, colon, prostate etc.

Both the above discussed technologies may be applied to HNC, where understanding the heterogeneity is the major concern. The technologies can be used individually or in a combinatorial approach (48) to first identify biomarkers and molecular targets specific to HNC and then to perform drug screenings/assays which will help in validating novel therapeutic agents and maximizing the success of a proposed therapeutic regimen in the patients (**Figure 1**). These techniques despite being very promising are limited by the lack of studies specific to HNC i.e. the literature is scarce. The review aims to encourage more such studies in the field of HNC research. This review also encompasses the omics profiles of single cell and is compared with bulk-cell analysis in HNC. We have discussed the single-cell derived spheroid based therapeutic advances and emerging targeted therapy that evolved due to omics studies. This review provides a panorama of the target landscape for the development of treatments of HNC. The gaps in the HNC treatment are being identified and future strategies to fill those gaps are suggested.

### Issues and Challenges in Bioinformatic Analysis With Reference to HNC

The omics analysis with reference of single cells, spheroids and organoids from HNC patient samples is a major challenge. One of the primary objectives of any omics analysis is to find reliable targets for therapeutic intervention. Such a task becomes possible with the identification of cell-specific genes which need to regulated specifically. Identification of biomarkers from a transcriptomics data typically start with the computational analysis of highly differentially expressed genes. This computational analysis becomes possible through well-established and benchmarked bioinformatics strategies, which face specific challenges in the case of single cell data emerging from HNSCC. Three issues require special attention viz. *(i)* much subtler changes in expression levels in single cell populations when compared to bulk expression data, *(ii)* sparsely collected data with lots of missing values, and *(iii)* absence of largescale relationships between single cell changes of expression and gene sets such as pathways or ontology terms, frequently used in interpreting bulk expression data outcomes.

To address the first of these issues (weak biomarker signal in differential expression), many computational tools, dedicated to the scRNA-seq data analysis have been developed (49, 50), which have allowed for significant advances in investigating heterogeneity and single-cell specific markers. A database of tools employed for scRNA-seq analysis has been reported and can be accessed *via* URL http://www.scRNA-tools.org (51). Tools like SCANPY (52) and Scatter (53) are some of the powerful robust pipelines that are well-integrated for comprehensive analysis (pre-processing and post-processing analysis) of scRNA-seq data. Many of these tools and resources provide expression data analysis of single cells, which takes into account the subtle gene expression level changes.

The second bioinformatics analysis issue is that of sparseness in the data sets. Poor coverage of expression values from each sample has two implications, (a) the very absence of the expression values may lead to missing the biomarkers altogether as only 10—20% values are reliably captured, (b) these dropouts adversely impact a confident grouping of cellular profiles into their subclasses as each transcript is described by a different set of genes. Few genes are present in one face while others are available in another. One of the solutions that has been proposed by bioinformaticians to address the sparseness of expression data in scRNA-seq is to reconstruct or predict the missing gene expression values, a process well-known as “imputation” in computer science. Traditional computational methods of imputation in general have dealt with a few or a small proportion of missing values in a data set. This problem is, however far more acute in the single-cell data due to much less information available to impute the missing ones. Imputing a missing value often relies on adopting a derived value from carefully selected similar samples. In single cell analysis, groups of samples are not known *a priori*. Hence, the question of identifying subclasses and imputing the missing values becomes a cyclic problem. Early computational techniques, developed for imputing gene expression values have included ZIFA (Zero-Inflated Factor Analyst) (55) and CIDR (clustering through imputation and dimensionality reduction) (56). Recently, SAVER, MAGIC and scImpute dedicated specifically to reconstructing a large number of missing values or imputations, were developed (57–59) and were successful in recovering the true expression of spike-ins transcripts improving and data quality.

Beyond the algorithms and tools for scRNA-seq analysis by addressing its sparseness, a number of data resources comprising transcriptomic and genomic information are also available in the public domain. The **Table 2** has listed the Gene Expression Omnibus (GEO) Dataset collection of transcriptome and associated data from HNC. Also, TCGA HNC dataset, which includes 527 cases, is a vast resource containing comprehensive integrative datasets of SNV, CNV, methylation, and slide images as well, complementing the transcriptome data. These datasets can be accessed from the GDC Data Portal (https://portal.gdc.cancer.gov/).

**Table 2:** List of GEO DataSets (expression profiling by array) related to HNC studies for data re- analysis.

S.No.	Title	Details	Platform	Series	#Samples
1	DNA methyltransferase inhibitor 5-aza-2’- deoxycytidine effect on oral cancer cell lines	Analysis of oral squamous cell carcinoma (OSCC) cell lines: OC3, SAS, SCC-15, and HSC3, treated with 5-aza-2’- deoxycytidine (AzC), an inhibitor of DNA methyltransferase. Results provide insight into potential tumor suppressor genes silenced by DNA hypermethylation in OSCC.	GPL6883	GSE38823	16
2	Oral squamous cell carcinoma- derived cell lines	Analysis of oral squamous cell carcinoma (OSCC)-derived cell lines. OSCC is a lethal disease with early death typically occurring as a result of local invasion and regional lymph node metastases. Results provide insight into the molecular mechanisms underlying OSCC.	GPL96	GSE31853	11
3	Squamous cell carcinoma of the tongue: tumor and histologically normal surgical margins	Analysis of oral carcinoma, histologically normal margins, and adjacent normal tissues from patients with squamous cell carcinoma (OSCC) of the tongue (training set). Results provide insight into molecular signature in histologically normal margins that are predictive of oral carcinoma recurrence.	GPL10526	GSE31056	96
4	Head and neck squamous cell carcinoma	Analysis of paired normal tissues and tumor samples from patients with head and neck squamous cell carcinoma (HNC). Results were used to assess the effectiveness of using a combinatorial approach to analyze microarray data in identifying differentially expressed genes.	GPL8300	GSE6631	44
5	Bleomycin effect on mutagen- sensitive lymphoblastoid cell lines	Analysis of mutagen-sensitive lymphoblastoid cell lines after exposure to bleomycin. Mutagen-sensitive cells exhibit a high number of bleomycin- induced chromatid breaks. Mutagen sensitivity (MS) reflects an individual's susceptibility to sporadic cancers. Results identify genes involved in MS.	GPL2902	GSE3598	28
6	Association between gene expression profile and tumor invasion in OSCC	Microarray analysis of cells obtained with LCM from 16 patients and compared these results with 4 control cell epithelium identified expression profiles differentially expressed between normal and tissues.	GPL96	GSE3524	20
7	Head and neck squamous carcinoma harboring papillomavirus	Analysis of 8 head and neck squamous cell carcinoma (HNC) tumor positive for human papillomavirus (HPV). 28 HNC HPV negative tumor examined. Between 15% and 35% of HNCs harbor HPV DNA. Results provide insight into the effect of HPV in HNC.	GPL570	GSE3292	36
8	Gene expression profiles of HPV - positive and - Negative Head/Neck Cervical cancers	Genome wide expression profiling of 84 HNCs, CCs and site-matched normal epithelial samples. LCM was used to enrich samples for tumor derived versus normal epithelial expression of a large subset of cell cycle genes was found to be upregulated in HPV+ HNC.	GPL570	GSE6791	84
9	450K analysis of 42 FFPE HPV+ and HPV- HNSCC samples	Methylation analysis of 21 HPV+ and 21 HPV- samples was performed.	GPL13534	GSE38266	42
10	Unique DNA methylation signature in HPV - positive Head and Neck Squamous Cell Carcinomas	Global and stratified pooled analysis of epigenome wide data was performed to identify tissue specific components and common viral epigenetic targets. Analysis revealed a novel epigenetic signature of HPV infection.	GPL13534	GSE95036	11
11	Gene expression profiling of archival tongue carcinoma and normal tongue tissue.	Total RNA was isolated from formalin fixed paraffin embedded (FFPE) samples. The expression data for 20818 genes was obtained using whole genome array.	GPL14951	GSE34105	78
12	Oral tongue cancer	To identify novel potential prognostic markers, 20 patients were grouped into stage (early vs. late) and nodal disease (node positive vs. node negative) subgroups and genes differentially expressed in tumor vs. normal and between the subgroups were identified.	GPL8300	GSE13601	58
13	Gene expression	Tumors with different HPV16 DNA and RNA (E6*I) status from 290 consecutively recruited HNSCC patients was compared by gene expression profiling and targeted sequencing of 50 genes. The study confirmed that the HPV16 DNA+ RNA+ tumors are HPV-negative (DNA-) HNSCC and have elevated expression of cell cycle genes and rare TP53 mutations.	GPL10558	GSE65858	270
				Total	794

The discussion above is based on the review of works on the issues of scRNA-seq analysis in general, which must anyway be addressed in HNC samples as well. However, so far there is only one published study that has specifically addressed the issue of single cell transcriptomics in HNC (60), while another study from the same group has comprehensively reviewed the bioinformatic approaches and key findings derived from for the single-cell technology-based study of cancer (61). These twin papers suggest that the HNC computational analysis and results can be broadly classified into three groups viz. (a) study of cellular heterogeneity and gene expression analysis (b) study of micro-environment of cancer cells and (c) process of invasion and metastasis of cancer cells.

Among the insights gained from bioinformatics analysis of HNC data sets at single cell levels, the foremost finding arguably is reported by Qi et al. (61) in which it was shown that patient outcomes under all treatment regimens are highly dependent on the intrinsic cellular heterogeneity. Intra-tumoral heterogeneity and tumor nest architecture was largely recapitulated within lymph node metastases. Specifically, it was observed that high heterogeneity measured by mutant-allele tumor heterogeneity (MATH) scores leads to poor patient outcomes, thereby highlighting the need to understand the cell population composition of HNSC cells to improve the patient survival rates. Authors also found that malignant cells’ expression patterns could not be distinguished from those of basal tumors, suggesting that most tumors could be defined as a single ‘malignant-basal’ cohort in OSCC. This contrasts with the glioblastoma multiforme (GBM) tumor, in which the malignant cells map to multiple different subtypes. These findings suggest that HNC tumor consists of lower diversity in malignant subtypes or because the subtypes have not been confidently resolved at this stage. Another study was performed to examine the change in tumor properties by simulating single-cell events leading to macroscopic tumor development. The model was able to successfully observe adhesion-driven cell movements and nutrition dependent heterogeneous tumor growth. Different treatment plans strongly influenced the final tumor cell type composition. The growth rate was observed to be significantly decreased when metabolism in tumor cells was upregulated. The mutation rates were adjusted, and low mutation rates cell types with higher division rate and delayed cell death started dominating the tumor. The models were also used to probe treatment regimens. Shorter pulses of chemotherapy were observed to have a better effect than a uniform application. The tumor size was significantly reduced by a single strong radiotherapy pulse as compared to multiple weaker pulses. The presence of tumor stem cells was confirmed to impact treatment outcome by increasing tumor size as well as heterogeneity. In view of the above results, the single-cell simulations can be a source of information to determine the heterogeneity and also predict treatment strategy and outcomes. These proved to be highly useful in improving the understanding of tumor development on a single-cell level but also the differences/similarities from bulk tumor analysis (62).

Since, cellular heterogeneity is so critical to HNC characterization and personalized treatment, researchers have tried to establish general patterns of cellular heterogeneity so prior therapeutics for each group can be developed. Bioinformatics work has concluded that the non-malignant cells from HNC patients could be grouped into eight main clusters by cell type viz. (a) T cells, (b) B/plasma cells, (c) macrophages, (d) dendritic cells, (e) mast cells, (f) endothelial cells, (g) fibroblasts, and (h) myocytes. However, the computational analysis so far has found that these non-malignant cells did not cluster as per their origin, when their expression profiles are used for automatic grouping, suggesting that the cell types and their expression states are consistent across tumor when their expression profiles are used for automatic grouping. On the other hand, malignant cells clustered well by the patient, suggesting expression changes across patients are more diverse than across cells of the same patient. In summary, malignant cells carry patient identity. The origin of cell from the 08 groups was not well encoded into the gene expression program. In the same context, another study by Yost et al. (63) on basal cell carcinoma using a combination of scRNA-seq and TCR sequencing, have implicated cancer-associated fibroblasts (CAFs) in tumorigenesis, tumor survival, ECM remodeling, immune system suppression, and tumor invasion system suppression, and tumor invasion. Another study by Leung et al. (64) focused on single-cell DNA sequencing, exome sequencing, and targeted deep-sequencing has investigated clonal evolution during metastatic dissemination in two colorectal patients. This study has highlighted that understanding the clonality at a single-cell level in a tumor is essential to simultaneously capturing and maintaining spatial information. Another study by Casasent et al. (65) has reported a method called Topographic Single Cell Sequencing (TSCS), which utilizes a combination of LCM (66) and single-cell DNA-sequencing to measure genomic copy number profiles of single tumor cells in breast cancer patients. This approach preserves single cells’ spatial context, which is critical to the location-specific therapeutic targeting strategies. Although the studies mentioned here are performed on cancers other than HNC, they successfully present strategies to combat the challenges associated with bioinformatics analysis.

Recently, a review published on the applications of single cell RNA sequencing in the field of otolaryngology, self-analyzed the single cell RNA seq data of HNC patients taken from the study by Puram et al. The analysis gave following findings that were relevant for clinicians 1) The scRNA-Seq data not only distinguished the disease causing cells from native tissue but also revealed the heterogeneity within diseased tissue samples. 2) Malignant cells from 10 HNC patients, when mixed, formed patient specific clusters i.e. with the cells of their original native tissue only. This suggested that clonal evolution is unique to each patient, and therefore the treatment strategy needs to be personalized. 3) Cells from the tumor microenvironment (TME) were also profiled along with malignant cells. However, these were not found to be clustering on patient-specific basis but rather on a cell-type basis. These cells could thus represent shared disease pathogenesis between all HNC patients that can be targeted using a similar therapy. 4) Rare cell types like stem cells, progenitor cells, CD4+ T-regulatory cells or exhausted T-cells were also identified from TME. These helped in understanding the disease maintenance, immune evasion and decreased efficacy of immune therapies. 5) Most importantly, the cell type specific biomarkers can be identified by investigating gene expression in heterogenous cell clusters detected by scRNA-Seq. For example, Puram et al. identified partial-EMT signature detected in a subset of malignant cells which was also present in existing bulk RNA-Seq tumor data. Such identifications can enable clinicians to determine the risks of nodal dissections on the basis of signatures indicating risk of metastasis. The prognostic signatures predicting survival, metastasis, chemoresistance can vary patient to patient. Such signatures can also be identified as markers to monitor drug response, emergence of resistance etc. before and after treatment. 6) Looking for genetic targets of FDA-approved drugs or small molecules in clusters of malignant sub-populations or TME cells can help identifying new druggable targets. A new database called Pharos describes 20,000 gene/protein targets and the drugs molecules available which can be further repurposed for use in HNC treatment (67).

Some bioinformatics studies have gone beyond biomarker discovery and cellular heterogeneity. Few researchers have used appropriate bioinformatics tools in creating and maintaining the tumor ecosystem’s spatial organization. Researchers have found that partial-EMT (P-EMT) cells were loosely arranged, and positioned in between malignant cells and CAFs. The study attributed the compactness of HNC tumor architecture to the expression of CD63 (68). Studies by Ligorio et al. (69) and Wagner et al. (70) in pancreatic and breast cancer respectively, have highlighted the need to utilize single cell separation method (SCS methods) with preserved spatial information, to gain insights into the role of intercellular interactions.

Another study by Navin et al. elucidated the tumor evolution process in breast cancer through sequencing of 100 single cells and revealed 3 distinct clonal sub-populations that represent sequential expansions. Contrasting to the gradual models of tumor progression their data indicated that tumors grow by punctuated clonal expansions. The study was performed on breast cancer and its liver metastases (71). More such studies on HNC will help in developing an understanding of the temporal progression of tumor heterogeneity. In response to systemic therapy, the issue of recurrence of tumor and overall temporal dynamics are other issues of transcription data analysis that heavily rely on suitable computational strategies, which are still under development.

### Limitations of scRNA-Seq in Clinical Medicine

The scRNA-Seq is a stride towards personalized medicine, but is still daunted by several challenges. Lack of large cohorts of scRNA-Seq data from human patient samples, high costs, user-friendliness, and tissue preservation are some of the major issues. The use of scRNA-Seq on individual patient tumors for drug selection is now feasible but more studies are still needed to establish personalized drug selection and drug repurposing using scRNA-Seq results for improved patient outcomes.

The cost of scRNA-Seq varies based on the chosen methodology, and hence depends on the cost of equipment, reagents, and sequencing. The costs of isolation and sequencing per cell have dropped significantly, but the throughput of sequencing machines has also increased, so the cost per run with more cells still remains high. Most of the platforms are available only in science laboratories and require a large investment and planning to procure for hospital use. In addition to cost, analysis of scRNA-Seq data requires basic bioinformatics knowledge and coding skills. Furthermore, standardization of different pipelines is also required for clinical use.

Tissue preservation is a major issue because of its fragility and cell viability. Currently, the use of frozen tissue samples or methanol fixed tissues for scRNA-Seq platforms is in its infancy. However, a few other options to aid tissue preservation are available and includes, temporary tissue stabilization buffers that can preserve cells for sequencing for 48 hours.

Generally, single nucleus sequencing (sNuc-seq) usually involves tissue disruption and cell lysis, carried out in cold conditions, followed by centrifugation and separation of the nuclei from the debris. It minimizes the skewing effect of degraded mRNA or cell-stress response genes on the data. Cell lysis in sNUC-Seq allows for potentially more efficient cell type delineation that includes for even the most interdigitated cell types. These advantages potentially make sNuc-Seq a better alternative to SCRNA-Seq. strategy.

## Omics of the Head and Neck Cancer

### Genomics of HNC

Single cell DNA (scDNA) sequencing is focused mainly on the copy number variations (CNVs) and identification of single-nucleotide variations (SNVs). These are the driving forces in biological processes which cause genomic heterogeneity and thus necessitate study of the cell at an individual level. The whole genome wide analysis of HNC identified mutations in many gene families, but the most significant percentage of mutations were observed in the *NOTCH* gene family (72–74), especially *NOTCH1*. *NOTCH* and many other known oncogenes, including cyclin E, *MYC*, and *JUN* are targets of *FBXW7*, a ubiquitin ligase. *FBXW7* is known to be mutated in 4.7% of cancers of HNC (74). Apart from this, more than 60% of mutations were observed in serine/phosphatidylinositol 3-kinase (*PI3K)* pathway genes such as *PTEN* and *PI3KCA* (75, 76). In fact, this is the most commonly affected pathway in HNC, and a more aggressive form of the disease can be attributed to multiple mutations in this pathway (77). Approximately 8-23% of HNCs possess mutation in *PTEN* that causes down-regulation and constitutive activation of threonine-specific protein kinase *Akt* and mammalian target of rapamycin (*mTOR)* (74, 78). It increases the susceptibility of the oral epithelium to carcinogens. The genome analysis in HPV positive HNSSC tumor showed mutations in *PI3KCA* gene leading to an increase in *mTOR* activity rather than Akt phosphorylation and hence helps explains the better efficacy of dual inhibitors against *PI3K/mTOR* (79). Interestingly, p53 was not found expressed in HNC tumors with *PTEN* downregulation, implying the exclusion of *p53* gene mutation (80).

**Figure 4:**
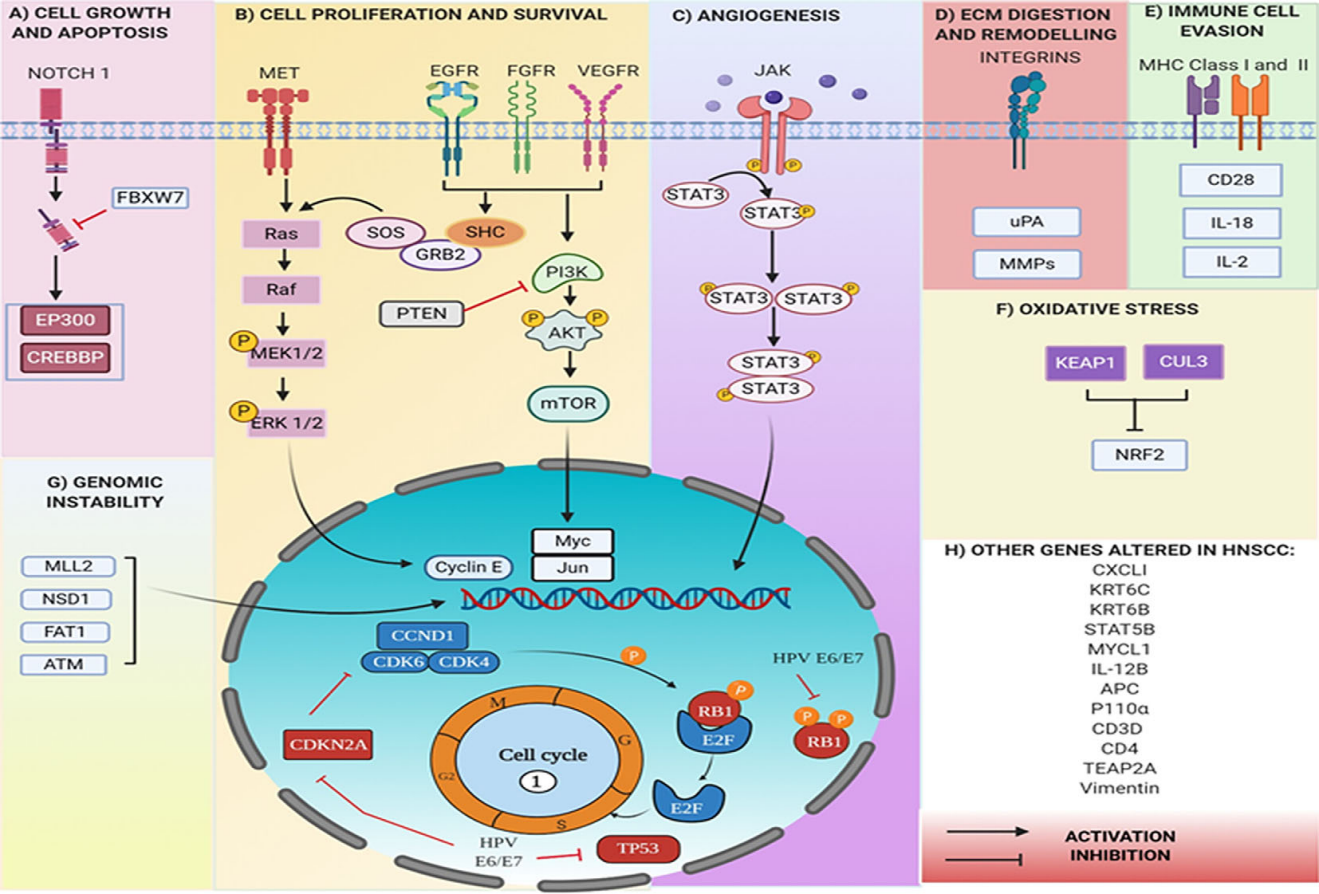
Genes altered in HNC at genomic and transcript levels. **(A)** Mutations in *NOTCH* gene pathway leads to cell growth and evasion of apoptosis, whereas **(B)** in *RTKs* (*VEGFR, EGFR, FGFR*) lead to alterations through *RAS/RAF/MAPK* pathway or *PI3K/AKT/mTOR* axis, eventually leading to uncontrolled cell proliferation, **(C)** in *JAK/STAT* pathway increase angiogenesis. **(D)** The integrins (*ITGA 3* and *ITGA 5*), *uPA*, and *MMP* 1,2,3,9.10,13 are all involved in ECM digestion and remodeling. **(E)** MHC I and MHC II expression is altered to evade recognition by immune cells. **(F)** Oxidative stress is increased due to mutations in genes like NRF2, whereas **(G)** mutations in *NSD1, MLL2, ATM* are characteristic of genomic instability. HPV proteins E6 and E7 inhibit *TP53* and *RB1*. All eventually leading to uncontrolled cell proliferation and **(H)**, multiple other genes are altered, producing significant effects.

The Epidermal Growth Factor Receptor (*EGFR*), a receptor tyrosine kinase (*RTK*) gene found upregulated in 80% of the patients suffering with HNC. The *EGFR* on activation causes cellular proliferation *via* either *RAS/RAF/MAPK* pathway, *JAK/STAT*, or *PI3K/AKT/mTOR* axis. Its over-expression in many HNSSC tumors is correlated to poor prognosis (81). In nearly 20% of HNC, oxidative stress genes are altered by mutation or variation in copy number. NRF2 (encoded by the *NFE2L2* locus) is a transcription factor that activates a cellular antioxidant response. It is overexpressed in 90% of the tumors leading to poor prognosis (82). Elevated *NRF2* levels are shown to cause chemoresistance in a variety of cancer cell lines that is reversible with siRNA inhibition of *NRF2* (83)⁠. Several chromatin-related genes in HNC *viz*, *MLL2* (a histone methyltransferase), *NSD1* (another histone methyltransferase), *EP300* (a histone acetyltransferase) and *FAT1* were also found to be repeatedly mutated in 19%, 10%, 7% and 23% of tumors respectively (84, 85). A recent study on HNSCC patients assessed the prognostic value of altered immune gene expression using a cohort of 96 patients (86). The expression of 46 immune-related genes was analyzed and, *4-1BB, IDO1, OX40L, GITR, FOXP3* were found significantly overexpressed along with *PD-1, TIGIT*, and *CTLA-4*. Almost half of the immune related genes had deregulated mRNA levels. The study assessed that a combination of high *OX40-L* and low *PD-1* mRNA levels, high *PDGFRB*, and low *CD3E* mRNA levels are associated with increased tumor recurrence. While *CD8A* was observed to be associated with poor prognosis, the increased expression of *PD-1* was associated with a good prognosis. These findings offer a therapeutic strategy in the treatment of HNSCC through the application of a combination of immune checkpoint inhibitors. Genetic alterations due to tobacco and betel quid chewing were also reported in oral cancer patients (87). These included i) single nucleotide polymorphism (SNPs) with non-synonymous type variations such as in *FAT1*& *2, TP53, NOTCH2*, Cadherin 3 (*CDH3*), and *ATM*; ii) synonymous type variations in Adenomatous Polyposis Coli gene (*APC*) (a tumor suppressor gene) and *IL12B* (cytokine gene). SNPs were also observed in non-coding regions, located in or near *EGFR, STAT5B*, Cyclin dependent kinase 5 (*CDK5*), and a protooncogene, *MYCL1* (**Figure 4**). Sayans et al. (88) analyzed 528 tumors of HNSCC subset in TCGA database and found 3491 deregulated genes. The somatic copy number alteration analysis showed *CDKN2A, CDKN2B, PPFIA1, FADD*, and *ANO1* as the most altered HNSCC genes. At the same time, genes with the most somatic mutations were *TP53, TTN, FAT1* and, *MUC16*. Another relevant result from the study was the mutual exclusivity pattern found between *TP53* and *PIK3CA* mutations. The difference in expression profiles between different studies i.e., the heterogeneity in the results could be attributed to the nature of the cancer.

### Transcriptomics of HNC

One of the recent applications of transcriptomics in cancer is the study of the cellular heterogeneity in tumor towards better understanding to achieve precision treatment. HPV positive HNC is a vital cancer type and has been identified with different gene expression patterns compared to HPV negative HNC. Transcriptomic data analysis between HPV positive and negative tumors provided important insights into the expression profiles (76, 89).Activated receptor *(RTKs)-RAS-PI3K* pathways and inactivated *TP53* and *CDKN2A* in HPV-negative tumors were observed. In HPV-positive tumors, *PIK3CA, FGFR3*, and *E2F1* were found to be activated while *TP53* and *RB1* were inactivated by viral oncoproteins *E6* and *E7* respectively. *PI3K* activation in HNC is reported by either of these mechanisms, receptor- tyrosine kinases, such as *EGFR* or mutation occurring in *PI3K* catalytic subunit, p110α (encoded by *PIK3CA* gene). Mutations often target one of two hotspot locations in the kinase or in helical domain, thereby promoting constitutive signaling through the pathway (90). Yu et al. (91) reported results from a network-based meta-analysis, identifying the biological signatures of HNC in pathways like integrin signaling, tight-junction regulation, antigen presentation, chemokine signaling, leucocyte extravasation, and vascular endothelial growth factor (*VEGF*) signaling.

Another transcriptomics study in HNC suggested the upregulation of genes involved in digestion and remodeling of the ECM, such as matrix metalloproteinases (*MMP*) 1-3, 9, 10, 13, urokinase plasminogen activator (*uPA*), Integrin alpha (*ITGA*) 3 and *ITGA5*. Both neoplastic and stromal cells secrete *MMP*s that digest certain components of the ECM (92) and promote cell migration and metastases in early stages of tumorigenesis (93, 94). Overexpression and activation of MMPs is critical in cancer progression and the pro-MMP-9/NGAL complex has been identified as a potential prognostic marker (95). A related study on a cohort of 145 oral cancer patients exhibited high levels of MMP2 in severe patients when compared to non-severe oral cancer patients. High levels of CD276 and low levels of *CXCL10* and *STAT1* were also observed to be associated with reduced overall survival. However, when compared MMP2 appeared to be a superior and independent prognostic marker (96).

The upregulation of interleukin (*IL*) 8, chemokine C-X-C ligand 1 (*CXCL1*), *CD28, CD3D, CD4, IL-18*, and IL-2 is observed in chemotaxis and lymphocyte activation while downregulation of *MHC* 1 &2 are hallmarks of invasive HNCs. Also, upregulation of VEGF and interleukin-8 (*IL-8*) connoted tumor cell angiogenesis, while *EGFR, STAT-3, PI3K*, and *NOTCH* upregulation influenced signal transduction pathways (97).

One hundred forty-six novel miRNAs expressed in HNC have been identified; but expression patterns among smokers and non-smokers remained undistinguishable. The three novel miRNAs significantly associated with HPV status, were mapped to chromosome 12 between genes Keratin 6C (*KRT6C*) and *KRT6B* (98).

Puram et al. have reclassified HNC into three malignant subtypes: classical, basal-mesenchymal and atypical. Single-cell transcriptomics from 18 HNC patients identified p-EMT as an independent predictor of grade, metastasis and critical pathological features (60). They performed the scRNA-seq analysis by considering 6,000 single cells from eighteen HNC patients containing five sets of matched primary tumor and lymph node metastases. The significant finding of the study was to distinguish among non-malignant (3363) and malignant (2215) cells on the basis of copy-number variations (CNVs) and epithelial cells where stromal and immune cells were excluded (60, 99, 100). Clinical and genomic meta-analysis of multicohort HNSCC gene expression profile has clearly demonstrated that HPV^+^ and HPV^-^ HNSCCs are not only derived from tissues of different anatomical regions, but also present with different mutation profiles, molecular characteristics, immune landscapes, and clinical prognosis. Cell lines and primary cells of HNC have been explored at single-cell transcriptomics (60, 101). The datasets have significantly improved the identification of distinct cells which are highly tumorigenic in nature in the HNC ecosystem. In the pool of cells, including malignant and non-malignant type, intra-tumoral variations at cell cycle, partial-EMT, proliferation, hypoxia-related genes have been observed. In this context, scRNA-seq is becoming a reliable technique for exploring HNC heterogeneity both at the genetic and functional levels. All the tumor influencing factors, such as circulating tumor cells (CTCs), immune cells, cancer stem cells (CSCs), present within, or in surroundings are investigated to gain clarity at a single cell level.

The scRNA-seq data may be used for understanding the drug response, as well as, drug resistance in individual HNC patients. The cetuximab-treated and untreated HNC cells yielded heterogeneous expressions of *TFAP2A* and *EMT* during the early stage of treatments, indicating onset of resistance. The expression variation analysis (EVA) analysis of scRNA-seq data suggests that cetuximab treatment increases cell heterogeneity, leading to evolution of different clonal cells with differentially activated pathways, thereby preventing EGFR inhibition (102).

A comprehensive multi-omics, single-cell analysis was performed in HNC cell lines by Kagohara et al., to identify responses to cetuximab, an anti-EGFR drug (102). It was observed that hundreds of genes altered their expression pattern as a response to the drug within 5 days of treatment. scRNA seq analysis identified onset of resistance following changes in various signaling pathways including regulation of receptor tyrosine kinases by Transcription Factor AP-2 (*TFAP2A*) and epithelial-to-mesenchymal transition (EMT) pathway. Different squamous cell carcinoma cell lines exhibited cell type dependent differential expression of *TFAP2A* and Vimentin (*VIM)* genes that corroborates inter cell line heterogeneity. The available HNC data bases provide clinical and genomic information on HNC cell systems (102–104). A holistic HNdb database curates all major omics data and literature on HNC-related genes (105). This database has laid the foundation for identification of possible biomarkers and development of HNC personalized medicine. It is interesting to note that a few genes are common in genomics, transcriptomics and scRNA-seq analysis of the HNC (**Figure 4**). These finding have stemmed from the independent studies. Therefore, it is imperative to perform integrated multi-omics studies and visualize molecular linkages using systems medicine for paving a way for personalized medicine.

The CSCs are responsible for failures of cancer therapeutics, drug resistance, and tumor recurrence. The single-cell transcriptomic data from salivary gland squamous cell carcinoma reported luminal and basal epithelial cells, as well as, small populations of CSCs. Overall, the study indicated that the process of tumorigenesis followed ‘gain-of-function’ by β-catenin and ‘loss-of–function’ by *Bmpr1A* mutations in basal cells, EMT markers expression, and activated Wnt signaling in CSCs of luminal cells (106).

### Proteomics of HNC

In order to minimize variables arising from HNC intra-tumor heterogeneity, analysis of differentially expressed proteins have been strategized. Bhat et al. (107) identified 286 biomolecules, having relevance in HNC. A few of these included i) insulin like growth factor binding protein (IGFBP) ii) downstream signaling components ERK, COX2, STAT, PFN2, EPCAM, SERPINH1, MCM2, iii) genes involved in prolactin signaling iv) angiogenesis v) DNA repair genes using integrated transcriptomics and proteomics approach. It has been reported that the ERK, COX2 and STAT1 proteins are important in progression and development of chemo resistance in HNC. Hence, these may be potential targets for effective therapy (108, 109). The saliva serves as a source for identification of bio-markers in cancer, and its proteomic analysis is considered to be a promising tool for HNC diagnosis; for example, over-expression of PLUNC and zinc-alpha-2-glycoprotein (110). To better understand the process of tumor progression and to make detection of cancer with precision, a technical triad of laser microdissection, protein chip technology and immunohistochemistry have been employed to identify the tumor relevant biomarkers. This study encompasses the protein profiling of calgranulin A and calgranulin B which are implicated in cancer pathology. Thus, such combinatorial approaches open up the possibility towards accurate prediction of metastasizing ability of a cell population (111, 112).

The proteins like Hsp90, VIM and keratin are already established bio-markers and drug targets while prelamin-A/C and PGAM1, have been recently suggested as potential markers (113). Bohnenberger et al. (114) identified distinct proteomic profiles between lung metastasis of HNC (metHNC) and squamous cell lung carcinoma (SQCLC). On classifying 51 squamous cell lung tumors, as either primary SQCLC or metHNC using proteomic approaches, 518 proteins with significantly different expression levels in HNC and SQCLC were identified. These proteins belonged to pathways involved in (i) vesicle transport, (ii) glycosylation, or (iii) RNA-processing. The FAM83H expression generally upregulated in cancers, was correlated to poor prognosis in HNC as well (115). The locoregional recurrence after chemotherapy (platinum-based concurrent chemoradiation) frequently occurs in HNC patients. It was observed that the intra-tumoral heterogeneity is linked to clonal evolution, and it is actually responsible for cisdiamminedichloridoplatinum (II) (CDDP) resistance in HNC (115). Niehr and co-workers (116) have applied targeted next-generation sequencing, fluorescence *in situ* hybridization, microarray-based transcriptome, and mass spectrometry-based phosphor-proteome analysis to elucidate the molecular basis of CDDP resistance. This resistance was observed to be associated with aneuploidy of chromosome 17, increased TP53 copy-number, overexpression of the gain-of-function (GOF) mutant variant p53R248L and increased activity of the *PI3K–AKT–mTOR* pathway, which were also considered as molecular targets for treatment optimization (116). Furthermore, label-free profiling of proteins in oral cancer has been performed by relative quantitation and employing nano-UPLC-Q-TOF ion mobility mass spectrometry hence, enabling rapid and simultaneous identification of multiple cancer biomarkers (117). This approach appears to have promising implications on tumor diagnosis. Single cell proteomics approach has encouraged system-wide protein profiling, direct assessment of immune cell health and tumor–immune interactions. This further helped augmenting evaluation of immunotherapy (118). Moreover, profiling of every single individual cell appears to indicate its role in tumor progression and molecular basis of the disease (119). The p53 tumor suppressor proteins have been counted in single colorectal cancer cells with 88% accuracy using the MAC chip (microfluidic antibody capture) (120). However, MAC chip utility in HNC is yet to be established. Multiplexing of protein markers at single-cell level using immunofluorescence methods have also been applied. However, single cell proteomics methods are in developing state and the proteome coverage is smaller in comparison to single-cell transcriptomics. In the context of precision medicine, integrating the protein based prognostic biomarkers is emerging as a supporting strategy for the treatment of cancer patients.

Most head and neck cancers expressing elevated levels of desmoglein 3 (DSG3) metastasize to the neck lymph nodes. The IHC and H&E reports may not always detect DSG3 during the initial metastasis process when metastatic lesions are less than 2mm in size. The use of sensitive methods like RT-PCR, scRNA-seq, and next-generation sequencing (NGS) is costlier and time consuming. Measuring the protein expression of tumor metastasis marker during the earlier phase of cell growth at the single-cell level for therapeutics provides additional advantages. The 3D printed microfluidics immune-array has a 10,000-fold higher sensitivity, which is superior to ELISA. This does not even requires any sorting experiments prior detection of proteins from a single cell. Not only it detects DSG-3, VEGF-A, and VEGF-C at lower concentrations, but its automated operations also provide results at a fast pace and lower cost. In addition to delivering information about HNC, it also quickly reproduces the results with minimal errors (121).

### Metabolomics of HNC

A comprehensive analysis of metabolites or metabolomic study is cardinal to cancer pathology as metabolome is a summary manifestation of all the other upstream omic profiles (122).

In a tissue metabolite profiling of HNC, 41 out of 109 metabolites screened were observed to be higher in tumorous *versus* non-tumorous tissues, while 15 appeared lower. Serum levels of glycolytic pathway metabolites increased (glucose, fructose, tagatose etc.), while that of several amino acids for example, lysine decreased significantly. Conversely, in tissue samples the glycolytic pathway metabolites decreased, and amino acids (valine, phenylalanine, threonine etc.) increased in tumorous *versus* non-tumorous tissues (122). Since, cancer cells depend more on aerobic glycolysis rather than oxidative phosphorylation for energy, and also use glutamine as major source of energy, they deplete glucose in hypo-vascular microenvironment. Also, amino acid levels are higher due to degradation of ECM in tumors. Another study showed the increased levels of polyamines in saliva of oral cancer patients in comparison to that of other cancer types. The choline to creatinine ratio revealed oral cancer specific elevation. In addition to this, 28 metabolites that accurately differentiate oral cancers from control samples were also identified. However, oral cancer may have higher impact on the metabolite composition of saliva in comparison to other cancers simply because of its location. Therefore, to confirm this a concurrent and comparative metabolic profile from saliva, blood and cancer tissue is warranted to confirm the oral cancer specific role of choline-creatinine ratio (123). Additional conformation was derived from another serum based study of 25 metabolites, of which 7 metabolites (leucine, isoleucine, taurine, valine, choline, tryptophan and cadaverine) were manifested in both the studies. Altered levels of urea and 3-hydroxybutyric acid were also reported for the first time in the later study (124).

A study by Wei (125), identified a signature panel of salivary metabolites (phenylalanine, valine, γ-aminobutyric acid, n-eicosanoic acid and lactic acid) whose levels were significantly altered in oral squamous cell carcinomas (OSCC). Hence these could potentially be used as biomarkers to distinguish between healthy and disease physiologies (125). While increase in lactic acid is simply explained by Warburg effect in glycolysis, valine and other amino acids are found significantly to be decreased presumably due to increased metabolic utilization. Increased ketone bodies, abnormal lipolysis, TCA cycle and amino acid metabolism have been reported in blood serum from OSCC patients (126). Patients with disease relapse exhibited increase in glucose, ribose, fructose, and tagatose with decrease in lysine, hippurate, trans-4-hydroxy-L-proline, and 4-hydroxymandelate in serum samples. A GC-MS based serum screening of OSCC revealed differences in 38 metabolites at pre-operative levels in comparison to healthy individuals. Furthermore, a comparison of pre-operative and post-operative metabolite profiles yielded significant differences in 32 metabolites. Seven potential biomarker candidates were found, i glyceric acid, lauric acid, N-acetyl-L-aspartic acid, ornithine, heptadecanoate, serine and asparagines. The sensitivity and specificity of biomarker pairs were assessed as 94.4% and 82.8% for ornithine+asparagine, 88.8% and 85.7% ornithine+glyceric acid, 88.8% and 97.1% ornithine+N-acetyl-L-aspartic acid, and 88.8% and 82.8% for ornithine+serine; endorsing their potential in early detection and stage identification in OSCC (127). An increase in choline compounds in OSCC implies its significant role in cancer feedback cell signaling. These increased choline levels renders it as a potential biomarker for cancer cell proliferation, survival and malignancy (128). Decreased levels of PUFA and creatine, and increased levels of amino acids and glutathione, were also observed in a study in tissues through proton high-resolution magic angle spinning magnetic resonance (HR-MAS MR) (129).

**Figure 5:**
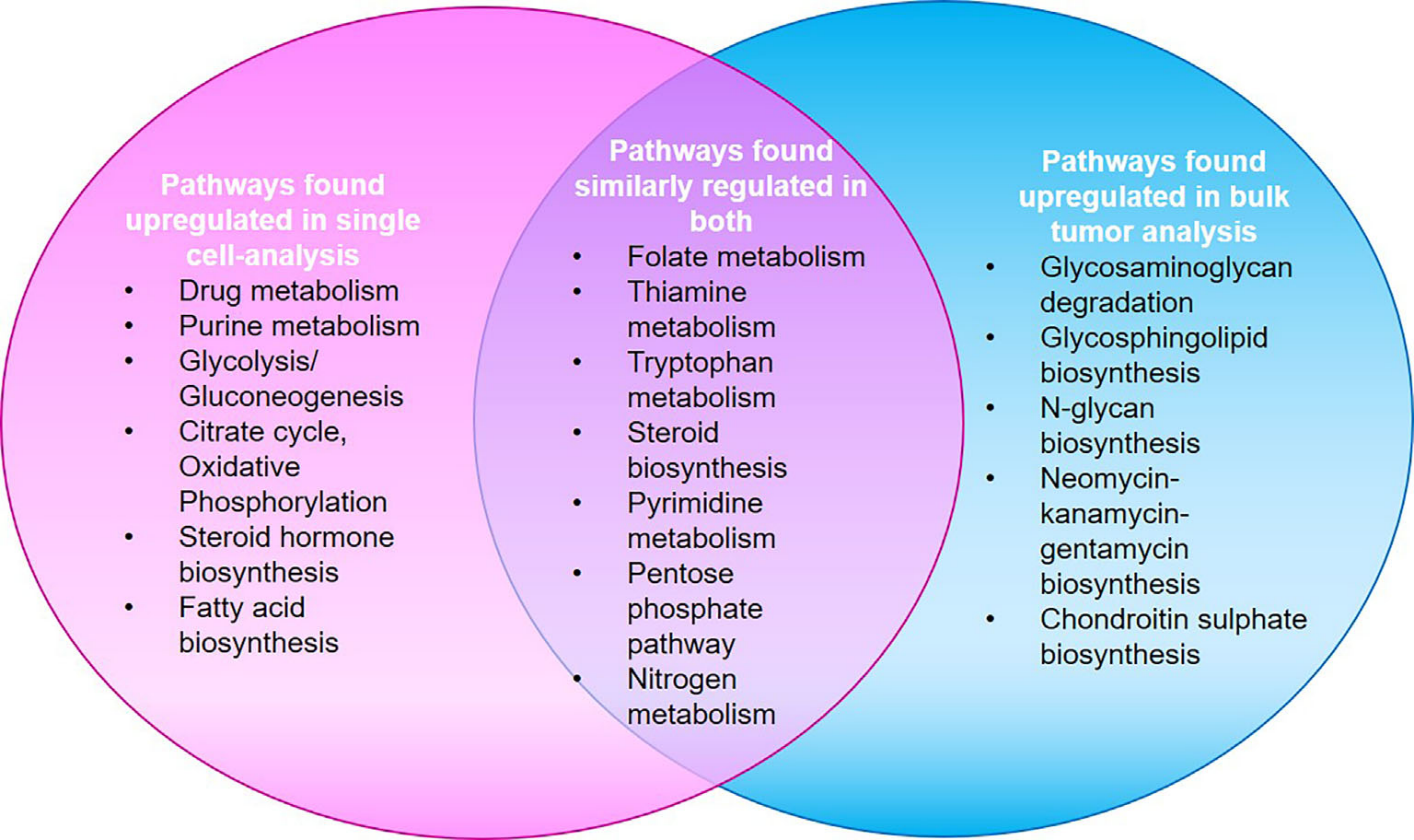
A study by Xiao et al. shows that major metabolic pathways found up-regulated in single cell analysis were found downregulated in bulk-tumor analyses and vice-versa. Twenty-four pathways showed similar up-regulation/down-regulation patterns in both as represented by the intersection in Venn (132).

The significant data on HNC metabolomics, is hindered by differences in detection and analytical methods. In addition, the inherent heterogeneity in HNC has obstructed the identification of an accurate biomarker for its early detection (130). Studies based on single cell analysis have shown significant differences from average pattern in bulk samples. Most metabolic changes in single malignant cells are not captured through bulk measurements as they tend to underestimate the highly complicated cellular composition of bulk samples. Though there is a universal upregulation of metabolic pathways, the over-expressions of certain genes (for example, OXPHOS i.e. oxidative phosphorylation pathway genes) are evidenced only at single cell level. Their absence at the bulk level is credited to the probable fallout of bulk measurements, enmeshed in the complexity of tumor composition. Differential expression from bulk level is also observed in genes involved in Vitamin b_6_ metabolism, lysine degradation, synthesis of aromatic amino acids, drug metabolism through cytochrome P450, degradation of fatty acids, oxidative phosphorylation, TCA cycle etc. However, where the expression at single cell and bulk level is different in purine metabolism, it was found similar in pyrimidine metabolism. Twenty-four out of fifty-six pathways show similar patterns of up-regulation or downregulation upon comparison between single malignant cells and bulk tumors, while 25 pathways that were reported downregulated through bulk tumor analysis were found upregulated on single cell level (131). **Figure 5** represents the major metabolic pathways upregulated in single cell and bulk tumor analyses. The inter-section in the Venn is indicative of pathways similarly upregulated or downregulated in both. The major cause of heterogeneity is the variations in mitochondrial metabolic activity (TCA cycle and Oxidative phosphorylation). Also, the metabolic features of immune and stromal cell sub-types were found distinct when the mean expression level of genes within these pathways were compared. Therefore, more single cell-based studies are required to not only gain better insights but also eliminate existing discrepancies, and to help identify different metabolic phenotypes in cell sub-populations.

### Epigenomics of HNC

Notably, intra tumor heterogeneity is the most significant hurdle in developing effective anticancer drugs, as targeted drugs and chemotherapy are effective until the development of drug resistance (133, 134). Tools like single-cell pharmacokinetic imaging have emerged as a powerful means to elucidate the mechanism of drug resistance in the tumor that may help overcoming the resistance (135). Characterization of cancer heterogeneity in epigenomic sub-populations appears to be relevant as cancer evolution, drug sensitivity, etc. are necessarily impacted by epigenetic alterations. This can be achieved using single-cell technology but is viable only at an early stage of cancer. In this context the degree of single cell chromatin accessibility also constitutes a significant challenge (136).

The epigenetic modifications are known to control programmed developmental changes and the ability of the genome to register, signal and perpetuate environmental cues (132). In order to sustain the inheritance of gene expression and biological functions, epigenetic mechanisms are linked to the transmission of cell lineage and phenotype from progenitor to progeny. These modifications are now known to be transmitted to the progeny cells with the epigenetic marks or genome bookmarking by transcription factors and other gene regulatory proteins (137, 138). The deviation from the transmission of normal epigenetic marking is suggested to be relevant not only in cell differentiation but also in the onset of several diseases, including cancer. In this context, some other vital chemical modifications altering chromatin states and subsequent gene expression patterns include DNA methylation, histone modifications, small non-coding RNAs, and chromatin remodeling factors. This is currently a subject of intensive study.

#### Methylation

Both, DNA and chromatin-associated proteins are modified to modulate DNA accessibility and chromatin structure (139). Methyltransferases like *DNMT3A* and *DNMT3B* are generally altered in malignancies (140). Abnormal expression of genes in many cancers is attributed to promoter-specific hyper-methylation for gene suppression, and genome-wide hypo-methylation (particularly in repetitive DNA) leading to gene upregulation (141, 142). DNA methylation is reported to affect most HNC genes involved in classical oncogenic pathways, cell cycle regulation (143–146), DNA repair (147, 148), *Wnt* signaling (149, 150), transmembrane proteins (151), tumor suppressors (152, 153), etc. **(** **Table 3** **)**. A recent gene comprehensive bioinformatics analysis using microarray data of DNA methylation and gene expression identified 27 aberrantly methylated genes with altered expression levels. *FAM135B* among them was hypomethylated and hence highly expressed. Multivariate cox proportional hazards analysis indicated that *FAM135B* could be a favorable independent prognostic biomarker for the overall survival of HNC patients (154). The primary risk factors like tobacco and alcohol use, human papillomavirus and Epstein-Barr virus infection can cause genetic and epigenetic alterations leading to the pathogenesis of HNC. Costa et al. (155) used TCGA data to identify distinct genetic and epigenetic particularities between HPV+ and HPV- HNSCC. The study primarily focused on gene promoter methylation patterns and was able to identify three different co-expression modules associated with HPV status. The genes were not only differentially expressed in HPV+ and HPV- cancers but also varied significantly between different stages of cancer. This indicated modulation of specific gene expression at different levels during cancer progression. However, a general pattern of expression (over or under) was observed throughout the stages (I-IV). Also, epigenetic modifications appeared pivotal for HPV infection as the association between methylation and gene expression was more potent in HPV+ cancers. *TP53*, *CDKN2A*, and *FAT1* appeared to be significantly mutated in HPV- cancers compared to the HPV+ ones. *CCNA1, PITX2*, *GJB6*, and *FLRT3* were found under-expressed and hypermethylated in HPV+ cancers while *SYCP2* was observed to be overexpressed in HPV+ oropharyngeal cancers. However, contrary to some reports, no association between *PIK3CA* and HPV+ cancers was observed in this study.

**Table 3:** The names and functions of genes modified epigenetically through methylation in HNC and their effects on development and prognosis of HNC.

S. No	Major classes of genes	Member	Function of genes	Epigenetic changes
1	Cell cycle regulation	p16	Inhibit CDK4 & CDK6	Hyper-methylation in promoter region leads to CDKN2A inactivation, frequent in HNC, de-regulation in cell cycle
		p15	Inhibit CDK	Promoter hyper-methylation in histologically normal epithelium of chronic smokers and drinkers
		TP53	Tumor suppressor	G to T transversions, patterns, differ between smokers and non-smokers
		CHFR	Early G2/M checkpoint.	Aberrant methylation, a potential biomarker
2	DNA repair	DAP-K	p53 dependent apoptosis	Hyper-methylation, a biomarker for early detection and prognosis
		RASSF1A	Pro-apoptotic, negative RAS effector	Inactivated TSG, promotes the development of cancer
3	Wnt signaling	ECAD	Formation of adherence junctions	Promoter hyper-methylation, loss of E-cadherin
		WIF 1	Secreted Wnt antagonist	Frequent methylation in OSCC, correlated with shorter survival of OSCC patients, possible prognosis marker
4	Transmembrane proteins	Cox-2	Prostaglandin synthesis	Increased expression, inverse relationship with E-cadherin
5	Tumor suppressor	DCC	Pro-apoptotic	Hyper-methylation in the promoter region in 75% of primary HNC
		KIF1A	Motor protein	Methylation of promoters is frequent in HNC, observed in 38% of salivary rinses

The putative role of gene promoter methylations or other epigenetic modifications provides favorable options for relevant therapeutic interventions. A study published in 2018 demonstrated an increased efficacy of immune therapy when combined with epigenetic therapy. The sensitivities of immune agents pembrolizumab and nivolumab were reported to be enhanced in a pre-clinical HNC model when combined with epigenetic drugs 5-azacytidine (DNA methyltransferase inhibitor) and romidepsin (histone deacetylase inhibitor) (156). Cancer specific DNA methylation patterns are effective early detection tools based on biomarkers generated from blood or epithelial cells shed in the lumen. The methylation status of 5 neuropeptide gene promoters (*SST, TAC1, HCRT, NPY*, and *GAL*) are also reported to be prospective alternative prognostic markers. For example, the methylation of *TAC1, HCRT* and *GAL* are indicative of poor survival in oral, laryngeal, and oropharyngeal cancers, respectively (157). The available information on methylated gene promoters is limited to a data subset, and the CpG island methylator phenotype (CIMP) is still under-investigated in HNC (158). Promoter hypermethylation has been observed in oropharyngeal cancers with HPV infection. A study by Esposti et al. (159), performed an epigenome-wide analysis using Illumina human methylation bead array data to identify differentially methylated CpGs associated with HPV infections. Five CpGs capable of predicting HPV status and survival were found in hypomethylated regions independent of anatomical site. This may help bypassing the issues associated with heterogeneity, arising due to different anatomies of HNSCC. It was observed that HPV has a genome-wide effect on the methylome that is independent of other risk factors. On the basis of DNA methylation patterns in 528 samples, 5 sub-clusters were identified. Of these pertained to HPV- cancers. Although 60% of differentially methylated genes were hypomethylated, the study also identified hypermethylation in genes *CDH18* and *CTNND2* that were found to be associated with HPV status. Promoter hypermethylation was also observed in *ZNF733*. The study not only highlighted hypomethylation of 60% genes for the first time but also suggested more pronounced effect of hypomethylation on gene expression than the hypermethylation. In addition, hypomethylation of many *cMyc* target genes was observed, and CpG island shore of *SYCP2* was found to be associated with increased gene expression. This observed role of *SYCP2* with another previously reported study (155). The 5 CpGs proposed as an epigenetic signature to identify HPV+ cases encompassed 3 genetic loci (B3GALT6-SDF4, SYCP2-FAM127B HTLF-HLTF-AS1). This predicted signature was able to integrate different epigenetic alterations and multiple exposure levels and hence this signature appeared as a better predictor of survival.

A study by Talukdar et al. (160) performed genome-wide DNA methylation profiling for esophageal squamous cell carcinoma (ESCC) using samples from 9 high incidence countries of Asia, Africa and South America. In the discovery phase, 108 tumors and 51 normal adjacent tissue while in replication phase 132 tumors and 36 normal tissues were analyzed. The study identified 6,796 differentially methylated positions and 866 differently methylated regions. Pathways important for cancer development like WNT and hippo signaling, cell communication pathways etc. were found enriched. *PAX9, SIM2, THSD4* were identified as top genes with crucial DNA methylation events, and were observed to be downregulated in tumors. Among all differentially methylated regions, 88% were found differentially expressed between normal and tumor tissues. The study also reported *THSD4*, *PHYHD1, GPT, KCNJ15*, and *TP53AIP1* for the first time in ESCC. However, there is ample scope for more such studies in HNSCC to identify non-random tumor specific methylation events to provide attractive avenues for biomarker development and therapeutic intervention.

#### Post-Translational Covalent Histone Modifications

Histone modifications such as acetylation, methylation, and ubiquitination of lysines, serine, threonine phosphorylation, etc., modify the accessibility of DNA for transcription factors and associated machinery. On comparing OSCC with healthy tissues, altered levels of histones *H3K4me2* and *me3* were observed (161). The significance of post-translational histone modifications can be understood by understanding their role in the development of chemoresistance which is also observed to be mediated by NFƘB. Studies have shown that chemo-resistant HNC cells have increased deacetylation of histones, that leads to chromatin compaction and further to impaired DNA damage repair. Subsequently, increased accumulation of histone γH2AX through serine phosphorylation, increases genomic instability. This implies chemoresistance may be prevented by HDAC inhibitors (162).

*SENP5*, a desumoylating enzyme, is overexpressed in OSCC and is related to poor prognosis (143). Likewise, lysine-specific demethylase 1 (*LSD1*) expression is upregulated in HNC, leading to increased growth and metastasis. Therefore, pharmacological attenuation of LSD1 should inhibit growth specific target genes and signaling pathways (161). Therefore, it is reasonable to speculate that epigenetic regulators and histone modulators might be alternative targets for the development of effective drugs for HNC.

#### Non-Coding RNAs

Non-coding RNAs do not code for proteins like RNA, but have enzymatic, regulatory, and structural functions (143). It is now known that microRNAs regulate cellular processes like proliferation, differentiation, and apoptosis *via* altered signaling in malignancies. Levels of miR-21, miR-16, and miR-30a-5p have been reported to be increased in HNC. Likewise, miR-205 and let-7a were also reported increased in both benign and malignant squamous epithelia (163). Conceivably, microRNAs act both as tumor suppressors or oncogenes. Epigenetic silencing of tumor suppressor mRNAs by CpG island hypermethylation is now emerging as a hallmark for human tumors. Hypermethylation in miR-148a, miR-34b/c and miR-9 was observed to be associated with downregulation of *CMYC, E2F3, CDK6* etc. (164).

A long non-coding RNA LINC00312 is significantly down-regulated in nasopharyngeal carcinoma. Since it inhibits the progression of the G1 to S phase, its reduced expression leads to tumor progression (165). HOX antisense intergenic RNA (HOTAIR) influences progression, metastasis and drug resistance in many cancer types. It is a prime candidate for a therapeutic target in cancer, as tumor cells contain significantly increased levels of HOTAIR, and its inhibition induces their apoptosis (166). The emerging understanding of HNC epigenetics is expected to benefit in understanding the prognosis and susceptibility of cancer to different therapies in isolation or their combinations.

The levels of complexity in epigenetic modifications have impeded their translation into instruments of cancer prognosis and therapeutics. Also, the bulk methodologies fail to capture the cellular diversity and tumor heterogeneity. Epigenome sequencing on single cell level can identify epigenetic and chromatin marks in single cells. A recent single cell based study identified the role of miR-142-3p in repressing *CLIC4*. *CLIC4* was found expressed more in tumor associated fibroblasts and endothelial cells as compared to tumor epithelial cells. The discrete patterns of localization and inverse co-relation of expression in both indicates the ambiguity related to bulk measurements (167). Development of advanced techniques like i) single cell genome-wide bisulphite sequencing (scBS-seq), ii) single cell chromatin integration labelling followed by sequencing (scCHIL-seq), and iii) single cell sequencing for transposable accessible chromatin (scATAC-seq) might provide insight into contribution of epigenomics in cellular heterogeneity. While these technologies uncover many aspects of cancer biology, further studies for HNC are still awaited. The applications of advanced techniques remains limited due to challenges in the unbiased amplification of a small amount of genetic material from a single cell (61). **Table 4** summarizes all the major biomarkers identified at bulk and single cell level. However, only limited studies are performed at single cell level, and therefore literature available is still limited. Therefore, reiterating the necessity of more studies at single-cell level to help remove discrepancies and facilitate accurate identification of biomarkers.

**Table 4:** Major biomarkers identified using different forms of omics profiling in head and neck cancer at single-cell level and bulk tumor level.

OMIC PROFILING	GENE/PROTEIN	SINGLE CELL ANALYSIS/BULK-TUMOR ANALYSIS
**Genomics**	NOTCH	Bulk tumor analysis
	FBXW7	
	PI3KCA	
	Akt	
	mTOR	
	EGFR	
	FGFR	
	VEGFR	
	NRF2	
	MLL2	
	NSD1	
	EP300	
	FAT1	
	TP53	
	CDKN2A/2B	
	PPFIA1	
	FADD	
	ANO1	
**Transcriptomics**	TP53	Bulk tumor analysis
	CDKN2A	
	E2F1	
	RB1	
	p110α	
	MMP	
	uPA	
	ITGA3/ITGA5	
	IL-8/IL-18/IL-2	
	CXCL1	
	CD4/CD28	
	MHC-1/2	
	KRT6C/KRT6B	
	VIM	Single cell based analysis
	TFP2A	
**Proteomics**	IGFBP	Bulk tumor analysis
	ERK	
	COX2	
	STAT	
	PFN2	
	EPCAM	
	MCM2	
	SEPINNH1	
	PLUNC	Saliva and serum
	Zinc-alpha-2-glycoprotein	
	Calgranulin A/B	Bulk tumor analysis
	Hsp90	
	VIM	
	Keratin	
	Pre-lamin A/C	
	PGAM-1	
**Epigenomics**	DNMT3A/3B	Bulk tumor analysis
	FAM135B	
	SENP5	
	HDAC	
	LINC00312	
	H3K4me2/me3	
	ΓH2AX	
	LSD1	
	miR-21/16/30a-5p	
	HOTAIR	
	TP53/TP53AIP1	
	CDKN2A	
	FAT1	
	CCNA1	
	PITX2	
	GJB6	
	FLRT3	
	SYCP2	
	CDH18	
	CTNND2	
	ZNF733	
	B3GALT6-	
	SDF4	
	HTLF/HLTF-AS1	
	PAX9,	
	GPT,	
	SIM2	
	THSD4	
	PHYHD1	
	KCNJ15	
	CLIC4	Single cell analysis
	miR-142-3p	

## Single Cell Derived Spheroids for Drug Assessment/Development

The current strategies for drug assessment and development involves the use of *in vitro* 2D techniques and animal models that are not only challenging in terms of genetic alteration and cellular heterogeneity but also are expensive approaches. The limitations of the 2D cultures are already discussed in *Heterogeneity: A Challenge in the Treatment of Head and Neck Cancer and Road Towards Solutions*. Single-cell and spheroid technology is an evolving science in HNSCC treatment, its therapeutic application comes in to play when selecting a chemical or biological agent. The gene expression patterns could be studied by using RNA sequencing from single cell derived spheroid, which can then be used to determine the most appropriate course of treatment on patient to patient basis. The data from recent melanoma studies suggests the presence of unique malignant cell signatures that are able to define the response to immune checkpoint inhibition (ICI), which is usually highly variable and difficult to predict, this could be a provocative possibility if extended to HNSCC (168). In other study an integrated analysis of cancer cells has been shown in HNSCC, where transcriptomes of ~6,000 single cells were profiled from 18 HNSCC patients to provide knowledge of the HNSCC ecosystem and define stromal interactions and a p-EMT process associated with metastasis, providing a detailed, molecularly-based predictor of adverse biologic features that drives clinical decision-making. Here, computational approach for inferring malignant cell-specific profiles from bulk expression data was used to refine HNSCC sub-types and provide a general scheme to extract information from other cancer datasets (60). Such study proves to be stepping stones in enhancing the understanding of intra-tumoral expression heterogeneity in epithelial tumors and might be able to guide future diagnostic strategies and treatment algorithms. Since even same type of tumor shows different response to the same therapy because of resistance and heterogeneity, it is important to identify the response of a tumor to any anti-cancer drug. The scRNA seq is powerful tool to investigate varying modes of chemoresistance in tumor cells derived from oral squamous cell carcinoma patients (OSCC). The cells isolated from the HNC patients undergoing cisplatin treatment were studied for drug resistance pattern, ITH, tumorogenic properties, and metastasis. Epithelial (ECAD+/VIM−) to mesenchymal (ECAD+/VIM+) transitions were identified in tumor and patient-derived cell lines. Also, it was determined that resistant cells can acquire metastatic characteristics and vice versa. The study highlights the predictive power of OSC7C patient derived primary cell line and scRNA-seq technology in revealing not only the course of tumor evolution in the clinic, but also in predicting mechanistic insight that can be exploited to design the next generation therapeutic strategies (169).

In another study, stem cell enriched 3D spheroid model was generated from cells taken from fresh tumor biopsies with different techniques such as hanging Drop (HD) and ultralow attachment (ULA) assays. The goal was to determine the ideal therapy regimen and identify mutation status specific to patients and therapy targets (170). In their approach, firstly the radiation treatment (2 Gy) plus cisplatin (2.5/5/10 μM) was given while in 2^nd^ approach chemotherapeutics alone were given. The study observed spheroids generated from ULA to be more reproducible and reliable than HD method. The spheroid model was found to be much better method for the study of drug effectiveness and mechanism behind drug resistance. But how the spheroids are developed are also important factor in drug screening and development. The two important spheroid growing techniques are culture free floating spheres (171) and multicellular tumor spheroid (MCTS) (172) which was earlier used for screening of several anti-cancer compounds. Both techniques have their own limitations. Thus, to screen the active compounds targeting cancer stem cells (CSC), stem cell-enriched spheroid model (SCESM) were generated using FaDu cells exploiting selective properties of both the techniques by Gorican et al. (173). Treatment of SCESM spheroid with all-trans retinoic acid (ATRA), a differentiating agent also used in HNSCC therapy reduced the stem cell marker expression, thus confirms the sensitivity and specificity of the spheroid.

In a study by Melissaridou et al. (174), cisplatin (1, 2 and 4 μg/ml) and cetuximab (60, 90 and 120 nM) treatment response were investigated on 3D tumor spheroids and 2D monolayers cells using a MTS-based assay. The cells cultured on 3D were found to be less sensitive to cisplatin compared to cells in 2D. The 3D spheroids were checked for the expression of three cancer stem cell (CSC) markers viz. CD44, SOX2 and NANOG and six EMT-associated genes (CDH1, CDH2, VIM, FN1, TWIST, FOXC2). A higher expression of CSC marker, CDH1 in 3D cultures was observed. EMT profile in HNSCC has been linked to drug resistance (175), however no evident pattern was observed in the study depicting towards other co-factors causing drug resistance.

Organoids developed by Driehuis et al. offered wide range of applications, which includes drugs screening of conventional drugs such as cisplatin, docetaxel, and fluorouracil or experimental targeted agents as well as predicting drug response of individual HNSCC patients. The study established starting point where chemo/RT response from multiple organoids generated from tumor biopsies of same patient can be directly compared to patient’s clinical response. Thus, establishment of an organoid model can lead to important advances in HNSCC diagnostics and treatment (36). In another study single-cell RNA sequencing was used in advanced melanoma to analyze sub-populations of T-cells. This study found significantly higher expression of TCF7 in treatment responders *versus* non-responders suggesting that the transcription factor TCF7 was among the chief markers predictive of a good clinical response (61, 176). As per the current scenario there is no similar multi-omics study, performed for HNSCC patients. Such studies could guide in analyzing changes in intra-tumoral heterogeneity with exogenous agents such as various forms of chemotherapy (e.g. cisplatin), biologic therapies (e.g. cetuximab), radiation, and ICI and could also help in patient selection for systemic chemotherapy or immunotherapy (61). From target identification to hit identification, single-cell spheroid has made its way as a new and emerging technique having significance at various levels in drug discovery. Although advancements in single-cell and spheroid technology are relatively encouraging, nonetheless there are no reports till date exemplifying validation and application of these technologies in clinical setups. Not much data is available on clinical application with patient-derived single cell spheroids and organoid in HNC. Hence, implementation and therapeutic application for the treatment of HNC in clinical routine is awaited.

## Potential Molecular Targets in Head and Neck Cancer

In cancer treatment, selection of therapy, drug administration, and dosing is a complex process varying on a case-to-case basis. The current treatment regimen used for HNC treatment aims at preserving organ and function, unlike the past treatment strategies. Though many targets are now being explored under different experimental set-ups for HNC treatment, the available drugs are against a minimal number of targets. In the past decade, several genetic mutation studies have identified specific essential genes that are mutated in the key biological pathways (**Table 5**) and could be potential targets for future drug development in HNC. The potential drug targets can be identified using integrated omics and mutational analysis to identify alterations in genes and pathways specific to HNC. Several mutational studies report most cancer-causing mutations in tumor suppressor genes instead of oncogenes (73, 90). Recently the targeted therapy (Precision Medicines) and gene therapy approaches have received a lot of interest from researchers. The targeted therapy approach takes advantage of differences between normal cells and cancer cells, interfering with specific “molecular targets” and blocking the growth and spread. The best known targeted therapies are Epidermal growth factor receptor (EGFR), monoclonal antibodies (cetuximab, panitumumab, zalutumumab, and nimotuzumab), checkpoint inhibitors (pembrolizumab and nivolumab), EGFR tyrosine kinase inhibitors (gefitinib, erlotinib, lapatinib, afatinib, and dacomitinib), vascular endothelial growth factor (VEGF) inhibitor (bevacizumab) or vascular endothelial growth factor receptor (VEGFR) inhibitors (sorafenib, sunitinib and vandetanib) and inhibitors of phosphatidylinositol 3-kinase/serine/threonine-specific protein kinase/mammalian target of rapamycin against HNC. On the other hand, gene therapy is an efficient anti-tumor treatment that uses genes to treat or prevent disease, and is rapidly evolving in cancer therapy (177). The in-depth transcriptomics and genomics studies could further determine the essential genes that could be considered for gene therapy in HNC treatment. The adenovirus vector carrying the p53 tumor-suppressor gene is one of the product for gene therapy approved in China for HNC treatment since 2003 (178). DNA damage response (DDR) pathway is another potential target for anticancer therapy. During the progression, most cancers lose one or more DDR pathways leading to greater genetic instability and increased dependence on other pathways. Targeting different proteins involved in the DDR pathway has shown efficacy in treating cancer.

**Table 5:** Alterations in key pathways in head and neck squamous cell cancer.

S. No.	Alterations of Pathway	Genes Involved	% Frequency of Mutations
1	Mitogenic	EGFR	3
		PIK3CA	20
		HRAS	3
		PTEN	2
2	Cell Cycle	CDKN2A	15
		Cyclin D 1(CCND1)	0
		Retinoblastoma (RB1)	3
3	NOTCH Signaling	F-Box and WD Repeat Domain Containing 7, E3 Ubiqutin Ligase (FBXW7)	5
		NOTCH1	19
		TP63	2
4	Oxidative Stress Response gene	KEAP1	4
		Cullin -3 (CUL3)	4
		NFE2L2	6

### Protein Kinases as a Drug Target in HNC

Protein kinases are involved in cancer metabolism and have been the second most important drug targets in the pharmaceutical industry after G-protein-coupled receptors. The crystal structures of kinase-inhibitor complexes of different families have been determined, these include (i) receptor tyrosine kinase (*EGFR*, *HER2*), vascular endothelial growth factor receptor (*VEGFR*), *JAK2, JAK3, Syk, ZAP-70, Tie2, EGFR, V-EGFR, FGFR*) (ii) non-receptor tyrosine kinase (Bcr-Abl) *NOTCH1*, Janus kinase (JAK) (iii) serine-Threonine kinase (*Clk, Dyrk, Chk1, IKK2, CDK1, CDK2, PLK, JNK3, GSK3, mTOR, p38 MAPK, PKB*) (iii) Rho-kinase (iv) Cyclin-dependent kinases (179). In these structures, active and inactive state of the protein kinases, ATPase pocket, point mutations, catalytic and non-catalytic domains of the kinases have been used as targets by kinase inhibitors and provided the mechanism of inhibition. A well-documented crystallographic analysis of the cAbl kinase domain with Gleevec inhibitor revealed locking of the protein kinase’s inactive conformation (180). The conformational flexibility and stability of protein kinases are central to their inhibition and subsequent drug designing strategies. IIdentifying the diagnostic significance of p38 isoforms in HNC and the subsequent design of specific peptide inhibitors against p38a MAPK aims to contribute to anti-kinase drug development, and the expanding expertise offers optimistic prospects for future cancer treatment.

#### ATPase Pocket of Protein Kinase as a Drug Target

The ATPase pockets of the protein kinases are quite conserved and offer an attractive target for drug design. It is important to understand whether a unique combination of specific amino acids or only a few conserved residues in ATPase site are involved in ATPase binding mechanism present in various protein kinases. The structures of protein kinase-A in complex with Fasudil and a more potent Rho-kinase inhibitor H-1152P were determined (182). The structural analysis shows the characteristic binding site within the ATP site, though the difference is of only two methyl groups between both the complexes.

#### Additional Binding Pockets of Protein Kinase as a Drug Target

An additional hydrophobic pocket close to the ATPase pocket in the protein kinase structure plays a crucial role in the inhibition mechanism. In p38 and C-Abl structures, a small threonine (Thr) residue lies as a gatekeeper and it interact with designed inhibitors to block ATP entry in the kinase domain. In the crystal structure of CDK2 with roscovitine (183), the isopropyl group of inhibitor interacts with gatekeeper Thr residue. In EGFR-tarceva crystal structure (184) the tarceva binds similar to aniline-quianzolines binding to CDK2 and p38 kinases (185), and acetyl moiety interacts with gatekeeper Thr residue. This pocket is similar to as observed in Gleevec-6-methyl group bound to C-Abl kinase.

#### Non-Catalytic Domain of Protein Kinase as a Drug Target

In protein kinase structures, non-catalytic domains have been observed, which play key role in kinase activity. The crystal structure of HER2 receptor in complex with herceptin Fab (186) shows that Fab binds at a specific site at the C-terminal of domain IV, which is involved in binding to other domains in closed conformation of HER1 and HER3 receptors. The Herceptin Fab binding to this pocket (close to the membrane) will engage the HER2 receptor with endocytosis machinery and inhibit the receptor signaling. In combination with radiotherapy, other EGFR antibodies IMC-C225 (cetuximab, Erbitux), Thermacin h-R3 (Cimaher) based on the given principle are getting quite successful in treating HNC cancer.

#### Single Residue in Protein Kinase as a Drug Target

Single residue in active sites of protein kinase plays a key role in inhibitor mechanism and can be used as a drug target. The BIRB796 (diarylurea) inhibitor binds to a specific sub-site in ATPase pocket of p38 kinase structure, incompatible for ATP binding (187). This BIRB796 inhibitor binds to Phe residue in the conserved DFG motif buried in a hydrophobic pocket located between two lobes of p38 kinase. In another case, from the structure of the complex between SU5402 and FGFR1 tyrosine kinase (188) the inhibitory binding modes of the indole-2-one family of anti-angiogenesis molecules (SU5416, SU668, SU1248) were identified. The methyl pyrrole ring attached to C3 in these inhibitors stabilized by an intra-molecular hydrogen bond between pyrrole nitrogen and the O_2_ atom of the oxindole ring. In SU5402-FGFR1 complex structure, a hydrogen bond between carboxyl group of SU5402 and the side chain of Asn568 of FGFR1 is important for inhibition.

Despite various encouraging results by kinase inhibitors, the critical challenges are drug resistance that mostly occurs through acquired resistance after initial treatment, toxicity, and compromised efficacy at the clinical level (189). In the clinical assessment, the key challenges are to develop efficient combinations of treatment after recognizing the kinase targets for particular cancer.

### G Protein-Coupled Receptors (GPCRs) as a Drug Target

G protein-coupled receptors (GPCRs) are involved in signaling pathways and can elicit both cytostatic and cytotoxic effects. Four of the GPCRs, (i) galanin receptor type 1 (GALR1) (ii) GALR2, (iii) tachykinin receptor type 1 (TACR1), and (iv) somatostatin receptor type 1 (SST1) are the most studied and promising therapeutic target in a wide variety of cancer. GALR1 & 2 both inhibit cell proliferation and apoptosis of HNC cells. GALR1 act through ERK1/2-mediated activation of cell cycle control proteins such as p27, p57, and suppression of cyclin D1 protein. In p53 mutant HNC cells, GALR2 was found to have anti-proliferative and pro-apoptotic effects (190). The significant reduced disease-free survival and a higher recurrence rate is associated with hypermethylation of GALR1, GALR2, TACR1, and SST1. Methylation of GAL, TAC, and SST and its investigation as potential prognostic markers in HNC has already been discussed in *Epigenomics of HNC*.

## Therapeutic Agents Targeting Potential Biomarker in HNC Patients

The primary treatment of HNC patients includes surgery, radiotherapy, and chemotherapy. However, a high recurrence rate, resistance to radiotherapy, and reduced life quality are major issues. Surgery and radiotherapy are the key treatments for early-stage tumors. However, therapeutic interventions are completely based on accessibility to the tumor, i.e., the tumor location, and not on the specific biology of the tumor. An increased understanding of cancer biology has led to the discovery of biomarkers, which can be efficaciously targeted to improve patient outcomes. Patients experiencing recurrence unable to be treated with surgery or radiotherapy, having limited overall median survival of one year, have shown better response to immune check point inhibitors targeting programmed cell death in HNC. Now clinicians need to determine that how targeted therapy can be best included/combined with immunotherapy. Clinical trials evaluating the combination of molecular targeted therapy with immunotherapy are emerging regularly. The results of such clinical trials will suggest us whether molecular targeted therapy and immunotherapy benefit different patients with different molecular alterations or can be used in combinations (191) (**Tables 6** and **7**)

**Table 6:** A List of Therapeutic agents and Their Mechanism of Action against HNC.

S. No.	Therapeutic agents	Clinical Phase	Mechanisms of Action
1	Cisplatin	FDA Approved	Interferes with DNA replication, kills carcinogenic cells. It acts through ERBB signaling pathways
2	Methotrexate	FDA Approved	Inhibits folic acid reductase, leading to inhibition of DNA synthesis and replication. It acts *via* interaction with enzymes of folate pathway.
3	5-fluorouracil	FDA Approved	Thymidylate synthase inhibitor. Inhibits deoxythymidine mono-phosphate (dTMP) production. dTMP is essential for DNA replication and repair and therefore its depletion causes cytotoxicity
4	Bleomycin	FDA Approved	DNA inhibition by induction of DNA strand breaks
5	Docetaxel	FDA Approved	Interferes with the normal function of microtubule growth by hyper-stabilizing their structure. It activates JNK signaling pathway and inhibits Hypoxia-inducible factor (HIF-α) and cancerous cell death under hypoxic conditions.
6	Pembrolizumab	FDA Approved	Targets programmed cell death protein (PD-1). PD-1, a member of the B7/CD28 family of co-stimulatory receptors, regulates the activation of T-cell.
7	Nivolumab	FDA Approved	Anti-PD-1 monoclonal antibody. It acts *via* inhibition of T cell proliferation and cytokine production
8	Cetuximab	FDA Approved	
9	Zalutumumab	Phase III	EGFR antagonist
10	Panitumumab	Phase III	
11	Pimotuzumab	Phase III	
12	AV-203	Phase I	
13	Afatinib	Phase I	Tyrosine Kinase Inhibitor (TKI). It targets the EGFR, HER2, and HER4
14	Poziotinib	Phase II	
15	Vandetanib	Phase II	Multitarget TKI. Targets EGFR and VEGFR
16	Nintedanib	Phase II	It targets VEGFR1–3, Platelet-derived growth factor receptor (PDGFR), and Fibroblast Growth factor receptor (FGFR1–2)
17	Gefitnib	Phase III	Selective, reversible inhibitor of EGFR tyrosine kinase domain
18	Erlotinib	Phase III	Inhibits the intracellular phosphorylation of tyrosine kinase of the EGFR
19	Lapatinib	Phase III	Inhibitor of the intracellular tyrosine kinase domains of both epidermal growth factor receptor and human epidermal growth factor receptor type 2
20	Dacomitinib	Phase II	Irreversible, potent inhibitor of HER1/EGFR, HER2, and HER4 tyrosine kinase.
21	EGFR antisense DNA	Phase II	Antisense DNA
22	Foretinib	Phase II	Target multiple RTKs
23	Figitumumab	Phase II	Human IgG2 mAb act against Insulin-like growth factor type I receptor (IGF-1R) pathway
24	Sunitinib	Phase II	Multi-target tyrosine kinase inhibitor
25	Sfatinib	Phase III	Potent, selective, and irreversible ErbB family blocker
26	Bevacizumab	Phase III	Anti **–**VEGF mab. It blocks the binding of circulating VEGF to its receptor.
27	SCH-58500	Phase III	
28	Advexin	Phase III	p53 stimulants (Recombinant adenovirus that encodes for gene human tumor-suppressor p53)
29	H-101	Phase III	
30	Gendicine	Phase II	
31	ONYX-015	Phase III	
32	Sorafenib	Phase II	Inhibit multiple intracellular and cell surface kinases in the Ras/Raf/MEK/ERK signaling pathways. The drug inhibits Raf-1, B-Raf, and kinase activity, PDGFR-β, VEGFR 2, hepatocyte factor receptor (c-KIT), and other proteins to inhibit tumor angiogenesis.
33	Dasatinib	Phase II	Inhibit multiple kinases
34	Buparlisib	Phase II	Inhibitor of PI3K signaling pathway (P13K inhibitor)
35	Alpelisib (BYL719)	Phase II	P110α inhibitor.
36	PX-866	Phase II	Inhibitor of PI3K pathway that binds to ATP catalytic site.
37	Copanlisib	Phase II	Inhibitor of class I PI3K (preferential activity against PI3Kα and PI3Kδ)
38	Temsirolimus	Phase II	
39	Everolimus	Phase II	mTOR kinase Inhibitor
40	Sirolimus	Phase I	*** ***
41	Gedatolisib	Phase I	class I PI3K and mTOR dual inhibitor
42	Ficlatuzumab	Phase II	Hepatocyte growth factor inhibitor
43	IRX-2	Phase II	multi cytokine stimulant that enhances the immune response. Akt/PI3K pathway is the prominent downstream target
44	INO-3112	Phase II	It is a combination of two previously developed DNA vaccines, VGX-3100 and INO-9012. It acts as an Immunostimulant.
45	MEDI4736	Phase II	Human IgG1 mAb that binds to Programmed cell death ligand 1(PD-L1) and blocks its binding to PD-1
46	Durvalumab	Phase III	mAb that targets PD-L1
47	MAGE-A3	Phase I	Peptide epitope vaccine. It elicits spontaneous cellular or humoral immune response.
48	MAGE-A3 HPV16 Vaccine	Phase I	Peptide epitope vaccine
49	DC vaccine	Phase I	Immunostimulant
50	Tavolimab	Phase I	IgG1 agonist antibody targeted against OX40 (a member of Tumor necrosis factor receptor family)
51	Urelumab	Phase I	Human IgG4 mAb that targets CD137 receptor
52	Utomilumab	Phase I	Humanized IgG2 mAb.Engages the immune costimulatory molecule 4-1BB/CD137
53	Ipilimumab	Phase I	Human mAb. It Blocks the interaction of cytotoxic T –lymphocyte antigen (CTLA-4) with its ligands (CD80/CD86)
54	Tremelimumab	Phase I	An IgG2 Ab. Involved in Immune activation by blocking the CTLA-4 negative costimulatory receptor.
55	Motolimod	Phase I	TLR-8 agonist that stimulates antigen-presenting cells that express TLR-8

The standard systemic treatment regimens for HNC include a combination of different drugs. However, overall survival rates are still very low, and due to the use of combinations of several drugs, the upper limit of toxicity seems to have been reached, causing the death of patients (192).

### Targeted Chemotherapy

The chemotherapeutic agents such as, afatinib, poziotinib, vandetanib, nintedanib, gefitnib, erlotinib, lapatinib, dacomitinib, alpelisib, PX-866 are the multitargeted inhibitors of protein kinases that regulate Ras/Raf/MEK/ERK/PI3K signaling pathways (193). The immunotherapeutic approaches such as specific antibodies targeting tumor, cytokines, cancer vaccines, and immune-modulating agents are other cancer treatment strategies, discussed below and in **Tables 6** and **7**.

A continuous flow of new molecules, explicitly targeting the upcoming biomarkers, is required as few of the promising agents have failed to show desired results in clinical trials. These include inhibitors of PI3K and mTOR pathways, e.g., px-866, an inhibitor of PI3K that binds to ATP catalytic site (194). Another antiproliferative and immunosuppressive drug sirolimus (extracted from streptomyces bacteria) demonstrated critical challenges in the form of poor bioavailability and long half-life in patients leading to frequent monitoring of the drug (195). Thus, substituting the drug with its better analogs with improved pharmacokinetic properties seems desirable.

A study by our group on the effects of a combination of two drugs against HNC showed that a combination of resveratrol and quercetin improved cytotoxicity and altered gene expression in oral cancer cells (196). The above combination of drugs was found to modify the epigenetic markers by downregulating histone deacetylases such as HDAC1, HDAC3, and HDAC8.

The cetuximab is an approved targeted therapeutic against HNC. It is accredited for first-line use with platinum-based chemotherapy: the chemotherapy plus cetuximab appreciably extended basic survival compared to chemotherapy alone. Significant improvements were visible within the progression-free survival and goal response prices. In a retrospective analysis of the trial, the enhancements observed with cetuximab were regarded on par with tumors being HPV positive against tumor being HPV negative (197). The single-cell analysis following treatment with cetuximab to different squamous carcinoma cell lines identified a heterogeneous cell population (198). Resistance to cetuximab appeared to be cell-type-specific which was attributed to altered gene expression of *TFAP2A* and *EMT*. However, resistance to cetuximab was found to be very common in HNC. Various evading mechanisms such as mutations in receptors may act in accordance to restore original oncogene dependence. A gain in copy number of target genes is another factor that counteracts the action of inhibitors. It has been found that altered copy number by amplification of chromosome 7p11.2 which encompasses EGFR gene, causes various cases of changes in EGFR activation in HNC (199). Gillison et al. (200) observed that with HPV-positive oropharyngeal squamous cell carcinoma (OPSCC) patients, cetuximab and radiotherapy demonstrated an inferior overall survival when compared with radiotherapy plus cisplatin.

The cisplatin plus fluorouracil treatments were given to 657 patients in the SPECTRUM phase III trial, with or without panitumumab, another monoclonal antibody targeting the EGFR receptor (201). A statistically non-significant trend indicated increased overall survival with the addition of panitumumab. As with the EXTREME trial using cetuximab, there was slightly more toxicity in the panitumumab arm than in the control arm. In the phase II trial (202) however, a comparison was made to identify the efficacy of panitumumab plus radiotherapy with chemoradiotherapy groups in locally advanced HNC patients. In the combined study, the efficacy of panitumumab was found to be inferior to cisplatin. It cannot be considered as its substitute for the treatment of unresected stage III–IVb HNC.

Larotrectinib is another type of targeted therapy that does not target specific cancer types but focuses on specific genetic changes in neurotrophic tropomyosin receptor kinase (NTRK) genes. This uncommon genetic change was found in head and neck cancer. *NTRK* is observed to be highly expressed in aggressive cancer and is used as a predictive biomarker and drug target (203). This FDA- approved TRK inhibitor is used for tumor-agnostic treatment after pembrolizumab (204).

Gene therapy is another targeted approach used to treat a variety of cancers, including head and neck cancer. The p53, a tumor suppressor gene, is mutated in over 50% of all types of cancers in humans. It plays a critical role in suppressing malignancy. Thus, restoration of functional wild-type p53 gene appears as a promising strategy for cancer treatment. The commonly used p53 stimulants are advexin, gendicine, ONYX-015, and H-101 (205).

### Monoclonal Antibodies as Targeting Agent

Monoclonal antibodies play a major role in the treatment of HNSCC. Monoclonal antibodies, such as antibody-drug conjugate to cytotoxic agents (206), are used to target particular cell surface proteins conferring tumor specificity by identifying selective targets. Clinically useful agents that target cell surface proteins in HNC such as AVID100 for EGFR; BAY1129980 for C4.4a; IMMU-132 target for TROP-2 antigen, and tisotumab vedotin are being developed and investigated (207–210).

The other approved targeted antibodies have been developed against specific targets such as CTLA-4 and programmed cell death protein 1(PD-1) that can stimulate co-stimulatory signals. The later one includes the agonistic mAb against OX-40 such as tavolimab and CD137 (urelumab, utomilumab) (211) or toll-like receptor 8 (TLR-8) agonist (motolimod) that mimics the viral ssRNA, the natural ligand of TLR8 and enhances immune response (212). The motolimod plus cetuximab plus was found to be safe in a phase I trial metastatic HNC patients (https://clinicaltrials.gov/ct2/show/NCT04272333) (213). The mAbs, pembrolizumab, and nivolumab are the approved PD-1 inhibitors. These have shown lasting responses in many cancers and were rapidly expanded for use in HNC treatments (214).

Another mAb that targets the EGFR domain, prevents a change in its conformation required for its activation. A randomized phase III trial with zalutumumab has failed to meet its endpoint of improved overall survival or no disease-specific survival and thus was suspended for further development (215). Other antibodies that targets different pathways/receptors and are under evaluation in head and neck cancer clinical trials. These include DLL/Notch pathway, FGF/FGF-R, HER2, TROP2 protein and VEGF/VEGF-R pathway and are discussed underneath.

Angiogenesis is important process in tumor growth and metastasis. The first-in-class anti-angiogenic mAb directed against ligand is bevacizumab that targets VEGF. Bevacizumab, has shown some evidence of activity combined with platinum-based chemotherapy. However, bevacizumab does not have a role in managing advanced or metastatic HNC outside of a clinical trial setting. Combining bevacizumab with chemotherapy in the first line of treatment of advanced metastatic HNC showed enhanced response rate and increased toxicity. In the E1305 trial, 403 patients without prior systemic therapy for advanced HNC were randomly assigned to platinum-based chemotherapy with or without bevacizumab (216). Thus, cisplatin with either fluorouracil or docetaxel or carboplatin with either fluorouracil or docetaxel were used. The primary endpoint was overall improved survival.

There has been substantial progress in the development of mAbs targeting FGFR pathway. Trastuzumab, a mAb targeting HER2, binds to domain IV of HER2 and blocks the homo-dimerization. In a phase II clinical trial study the effectiveness of trastuzumab on patients with advanced/metastatic salivary gland cancer was conducted, however no result is posted till date (NCT00004163) (217) (**Table 6**)

The transmembrane glycoprotein Trop2 is involved in several cell signaling pathways and is upregulated in a variety of cancers, including HNC. The overexpression of Trop-2 is associated with poor disease-free and overall survival in several solid tumors. IMMU-132 (hRS7-SN38 or Sacituzumab govitecan) is an Antibody Drug Conjugate (ADC) that target Trop-2. It consists of an antibody, hRS7 linked to SN38. SN38 is the active metabolite of irinotecan. The preclinical data demonstrated 136-fold more SN-38 delivery by IMMU-132 to a xenograft mouse model than irinotecan with lower toxicity, including lesser cases of severe diarrhea than irinotecan alone. IMMU-132 is under phase I/II clinical trials for evaluation of the safety and efficacy in patients of HNC (NCT03964727 & NCT01631552) (218–220). **Table 7** enlists clinical trials with combination of small molecules along with immunogens.

**Table 7:** Clinical trials of the combination of small molecules with immunogens against HNC.

Clinical trial number (NCT)	Therapeutic agent	Clinical Trial Phase
NCT02551159	Durvalumab (MEDI4736) ± tremelimumab *vs* standard of care (SOC) EXTREME regimen (cetuximab + cisplatin/carboplatin + fluoruracil)	III
NCT02369874	Durvalumab (MEDI4736) ± tremelimumab *vs* standard of care	III
NCT02741570	Nivolumab + ipilimumab *vs* SOC EXTREME regimen	III
NCT02952586	Avelumab+ cisplatin/RT *vs* cisplatin/RT alone	III
NCT03040999	Pembrolizumab + chemo/RT *vs* chemo/RT alone	III
NCT0276459	Cisplatin/RT± nivolumab	III
NCT02641093	Adjuvant cisplatin/pembrolizumab/RT	II
NCT02777385	Concurrent *vs* sequential pembrolizumab combined with cisplatin/IMRT	II
NCT02892201	Pembrolizumab	II
NCT03085719	Pembrolizumab with high *vs* high and low dose RT	II
NCT02823574	Nivolumab+ ipilimumab *vs* Nivolumab+ ipilimumab placebo	II

### Small Molecules as Targeting Agents

Small molecules have emerged as an important class of targeting agents that target multiple TK. The well-known molecular targets which have shown promising results are EGFR, EGFR TK and VEGF/VEGFR inhibitors and protein kinases or PI3K.

Gefitinib and erlotinib are the most common EGFR TKIs that are being used in clinical studies (phase II) for treatment of HNSCC. Lapatinib is another TKI that targets ErbB1/ErB2. The phase II study of lapatinib plus chemoradiotherapy in HNSCC has showed beneficial effect in HPV negative tumors (221). The lapatinib plus capecitabine combination has demonstrated best activity in the metastatic/recurrent HNSCC. Afatinib is an irreversible TKI that blocks the signaling originating from ErbB family. It is also used in other cancers with high EGFR mutations. In the stage III & IVa HNSCC, it is evaluated as adjuvant following radiotherapy (222).

Sorafenib, sunitinib and vandetanib are small molecules that targets VEGF (223). Sorafenib also act as radiosensitizer of HNSCC cells (224). Other VEGF inhibitors in clinical trials for treating HNSCC are linifanib, axitinib, pazopanib and nilotinib (225).

Several studies *in vitro* and *in vivo* demonstrated that temsirolimus, an mTOR inhibitor, inhibits proliferation of HNC. A study with HNSCC cell lines demonstrated beneficial effect of mTOR inhibitors plus cetuximab in the treatment of tumor with low EGFR expression or those that acquired resistance due to cetuximab/cisplatin (226). However, in other studies, temsirolimus failed to demonstrate significant changes in patients with advanced malignancies due to toxicity and subsequent death of patients. Everolimus, another mTOR inhibitor demonstrated antitumor effect in phase II clinical trial in patients with advanced HNSCC (NCT01111058) (227). The other small molecules that targets *BCR-Abl* kinase and are under clinical trial for treatment of HNSCC include imatinib, dasatinib, nilotinib, ponatinib inhibitors (193).

Although for the last several years, a large number of small molecules are being scrutinized against HNC (**Table 6**) with diverse heterocyclic structures, still a preferred specific and effective pharmacophore is yet to be assigned by drug development scientists. Current treatment is still associated with significant toxicities and includes chemotherapy mainly with platinum compounds, radiation, surgery, and a few targeted treatments. The scarcity of highly efficient drugs prompts researchers to identify novel targets for single-agent or for combined therapy.

## Conclusion and Future Perspectives of HNC Therapy

The major hindrance in the treatment of head and neck cancers comes with the associated heterogeneity. Organoid and single-cell technologies hold great potential in clinical translational research as they not only get the measure of this heterogeneity but also provide a means to encounter the problems it stems. The organoid technology has expanded to embrace genetic manipulation, various omics, drug-screening analyses, and diverse co-culture systems. In fact, multiple studies have shown similarities in patient responses and *in vitro* organoid studies. Single-cell technology, on the other hand, promises to identify and characterize alterations in sub-clone profiles. Given the rapid technology development in the field despite the remaining challenges, the combinatorial approach, including both these technologies, remains novel, innovative, and assuring in cancer treatment. The suitable assays for clinical implementation can be developed. Treating the model system with anti-cancer drugs may help distinguish responders from non-responders and hence, help find the right drug for the right patients potentially be leading to significant developments in the field of precision medicine. Multi-omics studies have shifted the focus on cancer driven perturbations at the whole cellular level. This helps identify molecular subtypes of the tumor, molecular signatures, and cellular responses at the clinic-pathological level based on a gene-protein expression. The multi-tiered approaches using the genomes, transcriptomes, and methylomes from carcinomas have aided our understanding of disease progression. However, integrating all the multi-omics data is crucial in identifying predictive signatures, i.e. integrating all molecular data and determining a minimal gene signature that distinguishes a tumor group. Patterns of alterations vary between patients as a result; it becomes essential to identify patient subsets with differential prognosis or the ones responding to different treatments (targeting therapies). The significant challenge still is the low availability of patient-derived models specific to head and neck cancers, the variability and diversity in treatment tested, and the absence of a standardized set of protocols to be followed. The clinical parameters tested vary inter-studies, and the quality needs to be ensured, primarily for drug screening assays. Also, the data available is limited mainly to Caucasian populations, while ironically, HNC constitutes 30-40% of total cancer cases in India. It reiterates the need for multi-omics based studies using organoid technology and single-cell analysis to identify unique biomarkers, drug targets, and signatures specific to Indian populations. The review aims to act as a compendium on the above technical advancements and their potential to identify biomarkers and test drug regimens.

## Author Contributions

RS, VT, and RT were involved in formulation and conceptualization and execution of the review article. Abstract and conclusion was written by RS. Introduction was written by NM and AT. Single cell technology, 2D and 3D technology is written by SM, RC, and JP. Genomics, transcriptomics & proteomics were written by PY and SK. Epigenomics & metabolomics done by RT and YJ. Potential drug target identification was done by AS and PY. PY and YJ compiled the manuscript, done referencing, creation and presentation of all tables and figures. The bioinformatic approach for data analysis was written by SA and SG. The coordination and management between authors was done by VT. Acquisition of the financial support for the project leading to this publication was done by RS, VT. All authors contributed to the article and approved the submitted version.

## Funding

We acknowledge the funding support from DST-DPRP [No. VI-D&P/546/2016-17/TDT(c)], BSR MID Career Award [No. F.19.226/2018 (BSR)] India for single cell-derived spheroid work in HNC.

## Conflict of Interest

The authors declare that the research was conducted in the absence of any commercial or financial relationships that could be construed as a potential conflict of interest.

## Publisher’s Note

All claims expressed in this article are solely those of the authors and do not necessarily represent those of their affiliated organizations, or those of the publisher, the editors and the reviewers. Any product that may be evaluated in this article, or claim that may be made by its manufacturer, is not guaranteed or endorsed by the publisher.

## Abbreviations

ASCs, Adult Stem Cells; HNSCC, Head and Neck Squamous Cell Carcinoma; ESCs, Embryonic Stem Cells; ECM, Extracellular Matrix; HNC, Head and Neck Cancer; HPV, Human Papilloma Viruses; iPSCs, induced Pluripotent Stem Cells; OSCC, Oral Squamous Cell Carcinoma; PDOs, Patient-derived Organoids.
